# Supplementary material for: Three-Dimensional (3D) in vitro cell culture protocols to enhance glioblastoma research
Source: PLoS One. 2023 Feb 8;18(2):e0276248. doi: 10.1371/journal.pone.0276248 (PMC9907841; doi:10.1371/journal.pone.0276248)
Supplement: S1 File — Also available on protocols.io. https://www.protocols.io/view/u-251mg-spheroid-generation-using-low-attachment-p-bszmnf46.pdf. (PDF) [file pone.0276248.s001.pdf]

U-251MG Spheroid generation using low attachment plate method protocol

Lara J Carroll[1], Brijesh Tiwari[1], James F Curtin[1], Janith Wanigasekara[1]
[1]Technological University Dublin

May 05, 2021

1

dx.doi.org/10.17504/protocols.io.bszmnf46

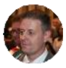

James Curtin
Technological University Dublin

ABSTRACT

3D cell culture is a process used to grow cells in vitro to mimic an in vivo environment.3D cell models are very useful for understanding disease mechanisms and exploring drug therapeutics. 3D cultures can be grown from cells taken from cancer organoids inpatients. Once grown, they can be used to screen for small molecule drugs or they can be genetically modified in order to analyse disease pathways or predict the toxicity or efficacy of a drug treatment. These cultures decrease the need to use animals in research and provides more reliable results as it uses human physiology.

This protocol describes the in vitro generation of spheroids using the low attachment plate method. This method uses low-adhesion plates that are coated with hydrophilic polymer to allow cells to cluster together, forming their own extracellular matrix, rather than sticking to the plate surface. The scaffold-free 3D cell culture models produced can more accurately reflect an in vivo microenvironment making them useful in the study of oncology, hepatotoxicity, neurology, nephrology and stem cell biology.

DOI
dx.doi.org/10.17504/protocols.io.bszmnf46
PDF
https://protocols.io/view/u-251mg-spheroid-generation-using-low-attachment-p-bszmnf46.pdf
HTML
https://protocols.io/view/u-251mg-spheroid-generation-using-low-attachment-p-bszmnf46.html
PROTOCOL CITATION
Lara J Carroll, Brijesh Tiwari, James F Curtin, Janith Wanigasekara 2021. U-251MG Spheroid generation using low attachment plate method protocol. **protocols.io**https://dx.doi.org/10.17504/protocols.io.bszmnf46

PROTOCOL STATUS
Working
We use this protocol and it's working

LICENSE
This is an open access protocol distributed under the terms of the Creative Commons Attribution License, which permits unrestricted use, distribution, and reproduction in any medium, provided the original author and source are credited

Citation: Lara J Carroll, Brijesh Tiwari, James F Curtin, Janith Wanigasekara U-251MG Spheroid generation using low attachmentplate method protocol https://dx.doi.org/10.17504/protocols.io.bszmnf46

This is an open access protocol distributed under the terms of the Creative Commons Attribution License (https://creativecommons.org/licenses/by/4.0/), which permits unrestricted use, distribution, and reproduction in any medium,

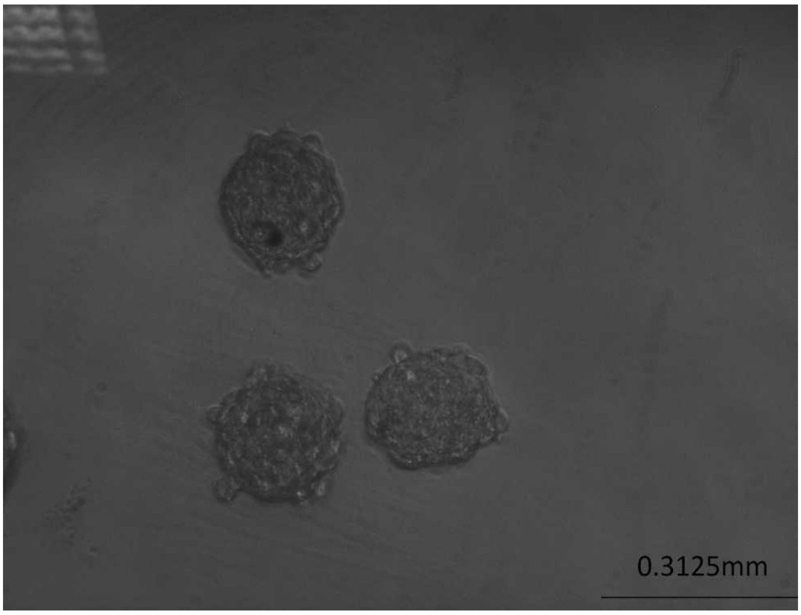

Figure 1: U-251 MG Human glioblastoma astrocytoma spheroids development using low attachment plate method (initial seeding density -10000 cells/ml). A) Tumorspheres growth after 2 days of incubation.

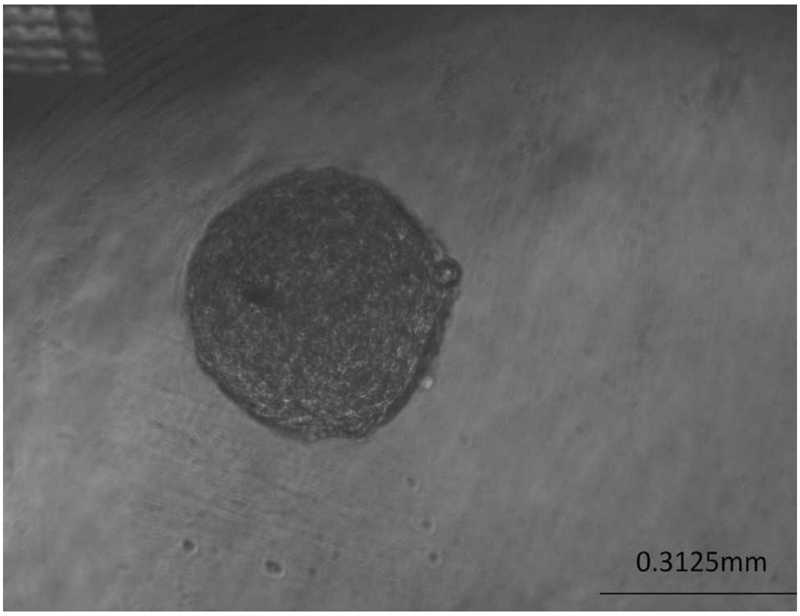

Figure 2: U-251 MG Human glioblastoma astrocytoma spheroids development using low attachment plate method (initial seeding density -10000 cells/ml). B) Tumorspheres growth after 4 days of incubation

Citation: Lara J Carroll, Brijesh Tiwari, James F Curtin, Janith Wanigasekara U-251MG Spheroid generation using low attachmentplate method protocol https://dx.doi.org/10.17504/protocols.io.bszmnf46

This is an open access protocol distributed under the terms of the Creative Commons Attribution License (https://creativecommons.org/licenses/by/4.0/), which permits unrestricted use, distribution, and reproduction in any medium,

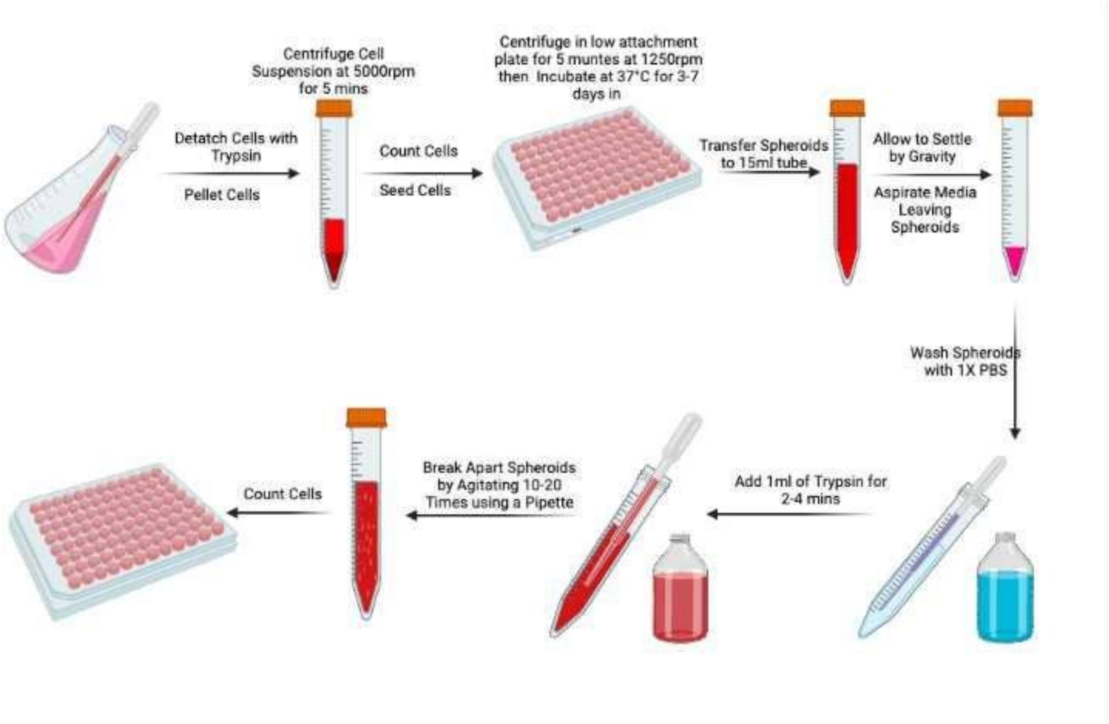

Figure 3: Graphic representation of using low attachment plate technique

GUIDELINES

This protocol is optimized for U251MG tumorsphere formation and this same protocol can be used for all different panel of cell lines with minor changes.

Some cell lines may require 15 minutes of incubation post-seeding and/or a pulse spin with different rotation speed and time in a centrifuge to allow cells to congregate more quickly before incubation.

Citation: Lara J Carroll, Brijesh Tiwari, James F Curtin, Janith Wanigasekara U-251MG Spheroid generation using low attachmentplate method protocol https://dx.doi.org/10.17504/protocols.io.bszmnf46

This is an open access protocol distributed under the terms of the Creative Commons Attribution License (https://creativecommons.org/licenses/by/4.0/), which permits unrestricted use, distribution, and reproduction in any medium,

MATERIALS

- Nunclon™ Sphera™ 96-Well (Thermo Fisher Scientific)
- Complete medium (Dulbecco's Modified Eagle Medium + 10% V/V Fetal Bovine Serum + 1% vol. of 100mM Penicillin-Streptomycin) [Sigma Aldrich]
- Mammalian cells (U-251 MG Human glioblastoma astrocytoma cells)
- Trypsin-EDTA solution(Sigma Aldrich)
- Centrifuge with swinging bucket rotor and well-plate holderCell
- counter or cytometer
- Centrifuge tubes
- Sterile reagent resevoirs
- Humidified incubator
- Bright-field microscope
- Phosphate Buffered Saline (PBS) 1X (pH41.7.4) [Sigma Aldrich]
- Trypan blue stain 0.4% (Thermo Fisher Scientific)
- Multi-channel micropipette and tips
- 100-1000 µl micropipette and tips
- Cell culture flasks and dishes
- Serological pipettes

Laboratory coat and gloves must be worn at all times

PROCEDURE

1 Prepare U-251MG single cell suspension at the desired seeding concentration. Cells can be from freshly prepared culture or from frozen vials in liquid nitrogen. Frozen vials can be immediately thawed using 37°C water bath and maintained in Nunclon Delta T25 cell culture flask with complete medium (Dulbecco's Modified Eagle Medium (DMEM) supplemented with 10% V/V Fetal Bovine Serum (FBS) + 1% vol. of 100mM Penicillin-Streptomycin for 1 passage before seeding for spheroid generation

2 Once flasks are 70-80% confluent, medium from the flask is aspirated and U-251 MG tumor cell monolayers are washed with 1X phosphate buffered saline (PBS; 5 ml for a 25 cm2 or 8 - 10 ml for a 75 cm2 flask), add 0.25 X Trypsin-EDTA cell dissociation enzyme (2 ml for a 25 cm2 or 4 ml for a 75 cm2 flask) and incubate cells at 37 °C for 2 - 5 min

3 Check cell detachment under a microscope and neutralize cell dissociation enzyme with complete growth medium. The 0.25 X Trypsin-EDTA reagent is neutralized using 4 volumes of complete medium

4 Centrifuge U-251 MG single cell suspension at 5000rpm for 5 min, remove supernatant, tap the tube and re-suspend cell pellet in complete growth medium using a pipette. This should yield a single cell suspension without cell clusters.

Citation: Lara J Carroll, Brijesh Tiwari, James F Curtin, Janith Wanigasekara U-251MG Spheroid generation using low attachmentplate method protocol https://dx.doi.org/10.17504/protocols.io.bszmnf46

This is an open access protocol distributed under the terms of the Creative Commons Attribution License (https://creativecommons.org/licenses/by/4.0/), which permits unrestricted use, distribution, and reproduction in any medium,

5 Cells with a viability >90% can be used for spheroid generation. Mix 10µl of cells with 10µl of Trypan Blue and count cells using a haemocytometer.

6 Calculated cell seeding density. Prepare a suitable volume of each cell seeding density to dispense the correct volume per well multiplied by the number of wells at each cell concentration.

7 Transfer the cell suspension to a sterile reservoir and dispense 200 µl/well into ultra-low attachment 96-well plates using a multichannel pipette ensuring pipette tips do not touch the surface of the wells to protect surface coating

8 Centrifuge the Nunclon™ Sphera™ 96-Well plates at 1250rpm for 5 minutes and transfer the plates to an incubator (37 °C, 5% CO_2, 95% humidity).

9 After 24h incubation, the media must be replenished. Remove 100µl media without disrupting tumorspheres and add 100µl of fresh media (DMEM + 10%FBS + 1%Ab) into each well and incubate at 37°C (5% CO_2, 95% humidity). The sides of wells should be used to remove or add media and pipetting should be carried out at average or below average speeds to avoid disruption to spheroids

10 U-251 MG Human glioblastoma astrocytoma spheroids will usually form in 3-7 days from the starting date depending on seeding density and visually confirm tumor spheroid formation daily using Bright-field microscope.

11 Imaging and quantification of spheroids can be also be monitored in situ using a cytometer and fluorescent live cell stains

Citation: Lara J Carroll, Brijesh Tiwari, James F Curtin, Janith Wanigasekara U-251MG Spheroid generation using low attachmentplate method protocol https://dx.doi.org/10.17504/protocols.io.bszmnf46

This is an open access protocol distributed under the terms of the Creative Commons Attribution License (https://creativecommons.org/licenses/by/4.0/), which permits unrestricted use, distribution, and reproduction in any medium,
