# Supplementary material for: Three-Dimensional (3D) in vitro cell culture protocols to enhance glioblastoma research
Source: PLoS One. 2023 Feb 8;18(2):e0276248. doi: 10.1371/journal.pone.0276248 (PMC9907841; doi:10.1371/journal.pone.0276248)
Supplement: S2 File — Also available on protocols.io. https://www.protocols.io/view/u-251mg-spheroid-generation-using-hanging-drop-met-btstnnen.pdf. (PDF) [file pone.0276248.s002.pdf]

U-251MG Spheroid Generation Using Hanging Drop Method Protocol

May 05, 2021

Lara J Carroll[1], Brijesh Tiwari[1], James F Curtin[1], Janith Wanigasekara[1]
[1]Technological University Dublin

2

dx.doi.org/10.17504/protocols.io.btstnnen

Technological University Dublin

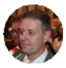
James Curtin
Technological University Dublin

ABSTRACT

The use of 3D cell culture has been a major step in developing cellular models that canmimic physiological tissues. Traditional 2D cell cultures are often unable to accurately represent the cellular functions and responses that are present in tissues, as a result, research findings based on 2D cultures tend to be skewed with limited predictive capability. 3D cell cultures can be grown from cells obtained from cancer organoids in patients. These models are useful for understanding disease mechanisms and exploring drug therapeutics in areas such as toxicity and efficacy.

In order to gather more physiologically relevant data, a variety of 3D cell culture techniques have been developed to mimic the in vivo characteristics of physiological tissues. This protocol describes in vitro generation of U-251MG spheroids using the hanging drop method. Advantages of using hanging drop plate method are, able to produce uniform size spheroids, low cost, comfortable to handling and suitable for short term culture. The main downside of this method is medium change, different drug treatment at different time points are impossible and labor intensive. This method uses the Perfecta3D hanging drop plate, a novel cell culture device that simplifies the process of spheroid formation, testing and analysis. Rather than having to invert the plates which often results in spillage or detachment, these plates are designed to create hanging drops using a plateau structure at the bottom of the plate.

DOI
dx.doi.org/10.17504/protocols.io.btstnnen
PDF
https://protocols.io/view/u-251mg-spheroid-generation-using-hanging-drop-met-btstnnen.pdf
HTML
https://protocols.io/view/u-251mg-spheroid-generation-using-hanging-drop-met-btstnnen.html
PROTOCOL CITATION
Lara J Carroll, Brijesh Tiwari, James F Curtin, Janith Wanigasekara 2021. U-251MG Spheroid Generation Using Hanging Drop Method Protocol. **protocols.io** https://dx.doi.org/10.17504/protocols.io.btstnnen

LICENSE
This is an open access protocol distributed under the terms of the Creative Commons Attribution License, which permits unrestricted use, distribution, and reproduction in any medium, provided the original author and source are credited

Citation: Lara J Carroll, Brijesh Tiwari, James F Curtin, Janith Wanigasekara U-251MG Spheroid Generation Using Hanging DropMethod Protocol https://dx.doi.org/10.17504/protocols.io.btstnnen

This is an open access protocol distributed under the terms of the Creative Commons Attribution License (https://creativecommons.org/licenses/by/4.0/), which permits unrestricted use, distribution, and reproduction in any medium,

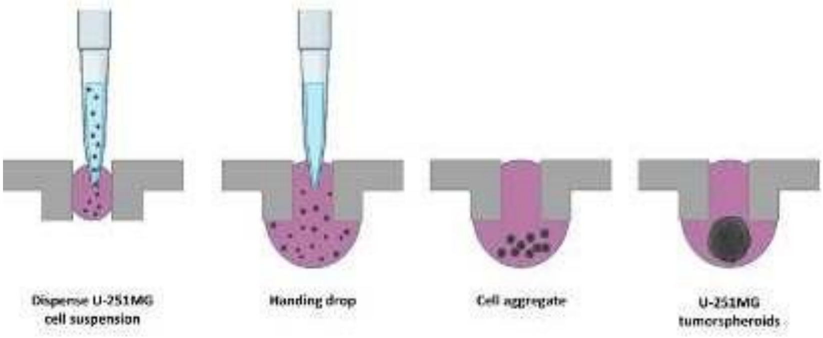

Fig. 1: Illustration showing main steps of hanging drop plate technique

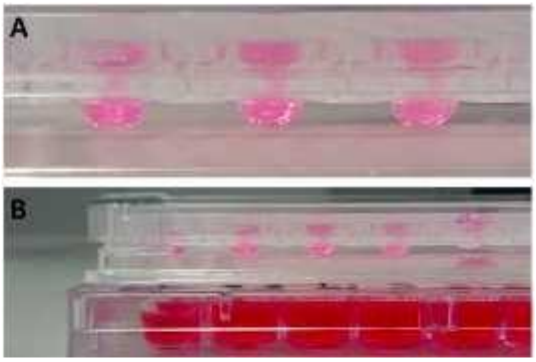

Fig. 2: Hanging drop plate. A) Hanging drops formation after adding 50µl of U-251MG cell suspension into the centre of each well from the top side of the plate. B) U-251MG tumorspherestransferring from the hanging drop plate to low attachment plates/pre-coated wells after 4 days.

Citation: Lara J Carroll, Brijesh Tiwari, James F Curtin, Janith Wanigasekara U-251MG Spheroid Generation Using Hanging DropMethod Protocol https://dx.doi.org/10.17504/protocols.io.btstnnen

This is an open access protocol distributed under the terms of the Creative Commons Attribution License (https://creativecommons.org/licenses/by/4.0/), which permits unrestricted use, distribution, and reproduction in any medium,

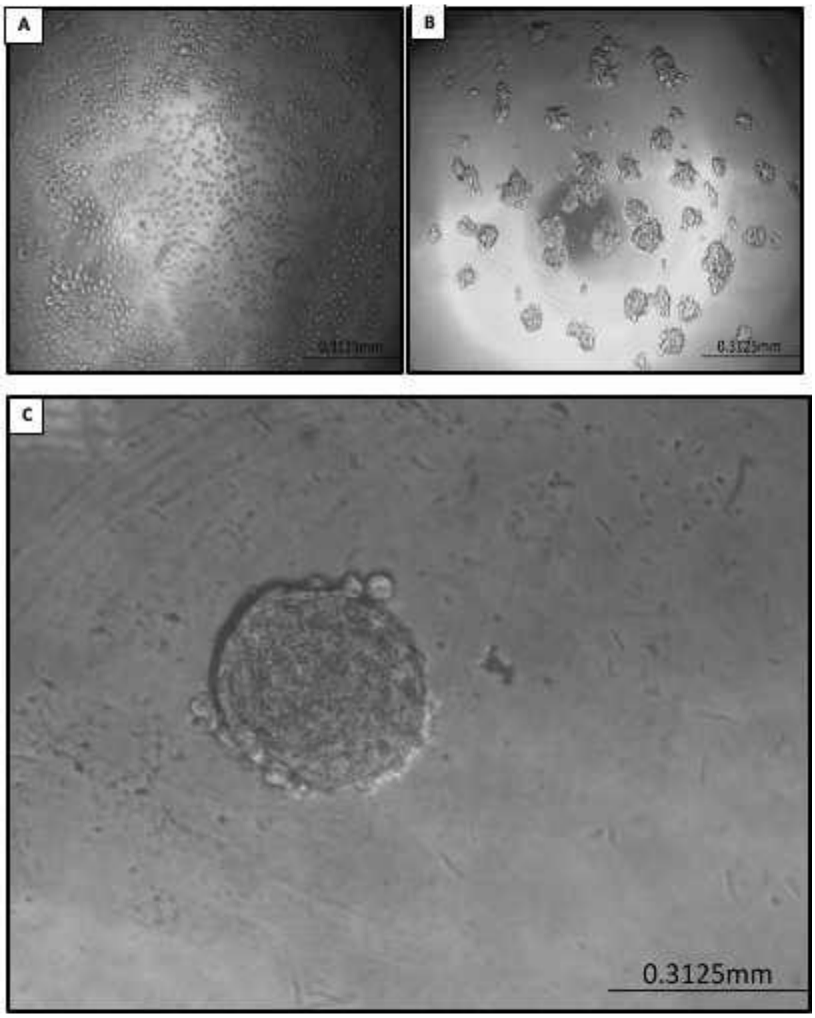

Fig. 3: Formation of U-251 MG tumorspheres by using hanging drop plate method. A) Cells in Day 0, B) Cell aggregates after Day 2, C) 3D spheroid after Day 4 of incubation.

Citation: Lara J Carroll, Brijesh Tiwari, James F Curtin, Janith Wanigasekara U-251MG Spheroid Generation Using Hanging DropMethod Protocol https://dx.doi.org/10.17504/protocols.io.btstnnen

This is an open access protocol distributed under the terms of the Creative Commons Attribution License (https://creativecommons.org/licenses/by/4.0/), which permits unrestricted use, distribution, and reproduction in any medium,

MATERIALS

- Mammalian cells (U-251 MG Human glioblastoma astrocytoma cells)HDP1096
- Perfecta3D® 96-Well Hanging Drop Plate (Sigma Aldrich)
- Complete medium (Dulbecco's Modified Eagle Medium + 10% Fetal Bovin Serum + 1% Penicillin-Streptomycin (Sigma Aldrich)
- 1X Phosphate-buffered saline (PBS) (Sigma Aldrich)
- Trypsin-EDTA solution (Sigma Aldrich)
- Multi-channel micropipette and tips
- 100-1000µl pipette and pipette tips
- Bright-field microscope
- Humidified incubator
- Centrifuge with swinging bucket rotor
- Centrifuge tubes
- Cell counter or cytometer
- Sterile reagent reservoirs

Laboratory coat and gloves must be worn at all timesUse laminar flow hood when working with cultures

PROCEDURE

1. U-251 MG cells are cultured in 100mm or 150mm dishes until reaching approximately 80-90% confluency and washed with 1X PBS solution

2. Dissociate U-251 MG cells by incubating for 4 minutes at 37°C with Trypsin-EDTA solution (0.25%w/v) and inactivate the trypsin by re-suspending in the full growth medium pre-warmed in a 37°C water bath

3. Centrifuge U-251 MG cell suspension for 5 minutes at 300g, re-suspend the cell-pellet in 1ml offull growth medium and pipette cell suspension up and down thoroughly to obtain a uniform single cell suspension

4. With a pipette, add sterile, room temperature PBS to the reservoirs located on the peripheral rim of the plate and tray which are divided into sections. Add 2ml per plate reservoir section and 1ml per tray reservoir section. This prevents the hanging drop from drying out during incubation. Note: Take care to not tilt the plate if filling with water as this may result in spillageand contamination of hanging drops

5. Prepare a 10ml stock solution of U-251MG cell suspension in full growth medium at a concentration of $2.5x10^5$ cells/mL in order to achieve 5000 U-251MG cells per 20µl hanging drop. (Each hanging drop well is able to hold 20-50µl cell suspension and any volume above50µl will result in drop instability). Mix the cell suspension thoroughly by gently pipetting upand down using a 1000µl pipette to ensure all cells are fully suspended within the media

6. Hanging drops can be formed by carefully pipetting 20-50µl of cell suspension into the centreof each well from the top side of the plate. Use a multi-channel pipette but only add 2-4 dropsat once as multiple seeding can affect the accuracy and placement of the drops. Hanging drops should be formed on and confined to the bottom of the plate

Citation: Lara J Carroll, Brijesh Tiwari, James F Curtin, Janith Wanigasekara U-251MG Spheroid Generation Using Hanging DropMethod Protocol https://dx.doi.org/10.17504/protocols.io.btstnnen

This is an open access protocol distributed under the terms of the Creative Commons Attribution License (https://creativecommons.org/licenses/by/4.0/), which permits unrestricted use, distribution, and reproduction in any medium,

7. Place the lid on the plate and insert the assembly into a humidified cell culture incubator at 37°C and 5% CO_2. After a couple of hours, individual cells should begin to aggregate and form into spheroids. Time taken for spheroid formation differs between cell types but is typically a minimum of 4 days. U-251MG tumorspheres will form within 4 days and formation can be confirmed visually and monitored daily using a bright-field microscope. NOTE: Extra care must be taken not only when transporting the plates to/from the incubators, but when opening and closing the incubator in general as excessive movement can cause the plates to shift and the spheroids to either fall or form incorrectly

8. Cell culture media should be exchanged on a daily basis to provide enough nutrients for cells and to prevent osmolality shift of the media. Add 5μl of fresh media back into the hanging drops by placing the end of the pipette tips in the neck region of the access holes/wells and slowly dispense fresh media into the access holes

9. Once formed, tumorspheres can be transferred from the hanging drop plate to low attachment plates/pre-coated wells in the dish by adding 50μl of fresh media into each hole

Citation: Lara J Carroll, Brijesh Tiwari, James F Curtin, Janith Wanigasekara U-251MG Spheroid Generation Using Hanging DropMethod Protocol https://dx.doi.org/10.17504/protocols.io.btstnnen

This is an open access protocol distributed under the terms of the Creative Commons Attribution License (https://creativecommons.org/licenses/by/4.0/), which permits unrestricted use, distribution, and reproduction in any medium,
