# Supplementary material for: Three-Dimensional (3D) in vitro cell culture protocols to enhance glioblastoma research
Source: PLoS One. 2023 Feb 8;18(2):e0276248. doi: 10.1371/journal.pone.0276248 (PMC9907841; doi:10.1371/journal.pone.0276248)
Supplement: S3 File — Also available on protocols.io. https://www.protocols.io/view/u-251mg-spheroid-generation-using-a-scaffold-based-bszqnf5w.pdf. (PDF) [file pone.0276248.s003.pdf]

U-251MG Spheroid generation using a scaffold based method protocol

Lara J Carroll[1], Brijesh Tiwari[1], James F Curtin[1], Janith Wanigasekara[1]

[1]Technological University Dublin

May 07, 2021

1

dx.doi.org/10.17504/protocols.io.bszqnf5w

Technological University Dublin

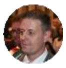

James Curtin
Technological University Dublin

ABSTRACT

3D cell culture is a technique that is used to grow cells in vitro that will mimic an in vivoenvironment. 3D cell models are a helpful learning tool for researchers to better understand disease mechanisms and to explore different therapeutic properties of drugs. 3D cell cultures can be developed using patient derived cancer cells. Once they have been grown, these 3D cells can be used to screen for small molecule drugs or for genetic modification in for analysis of disease pathways or to predict drug treatments toxicity or efficacy. 3D cell cultures are a big step towards the more ethical testing of drug toxicity and efficacy as they decrease the need to use animals in research as wellas providing more reliable results as the cells used are of human physiology.

Cellusponge are 3D porous hydroxipropylcellulose scaffolds that are designed for use with cells that do not require specific ligands. As well as the standard non-coated cellusponge, there are two more of the same type of scaffold available for use that aremade with two different coatings to allow for improved adaptation of different cell types, these are called Cellusponge-Gal and Cellusponge-Col. Cellusponge is a no- coating approach that is intended for use in the development of general soft tissue 3D culture. It has been used as soft matrix for 3D cell culture and 3D tumour model.

DOI
dx.doi.org/10.17504/protocols.io.bszqnf5w
PDF
https://protocols.io/view/u-251mg-spheroid-generation-using-a-scaffold-based-bszqnf5w.pdf
HTML
https://protocols.io/view/u-251mg-spheroid-generation-using-a-scaffold-based-bszqnf5w.html
PROTOCOL CITATION
Lara J Carroll, Brijesh Tiwari, James F Curtin, Janith Wanigasekara 2021. U-251MG Spheroid generation using a scaffold based method protocol. **protocols.io**https://dx.doi.org/10.17504/protocols.io.bszqnf5w

LICENSE
CC BY This is an open access protocol distributed under the terms of the Creative Commons Attribution License, which permits unrestricted use, distribution, and reproduction in any medium, provided the original author and source are credited

Citation: Lara J Carroll, Brijesh Tiwari, James F Curtin, Janith Wanigasekara U-251MG Spheroid generation using a scaffold basedmethod protocol https://dx.doi.org/10.17504/protocols.io.bszqnf5w

This is an open access protocol distributed under the terms of the Creative Commons Attribution License (https://creativecommons.org/licenses/by/4.0/), which permits unrestricted use, distribution, and reproduction in any medium,

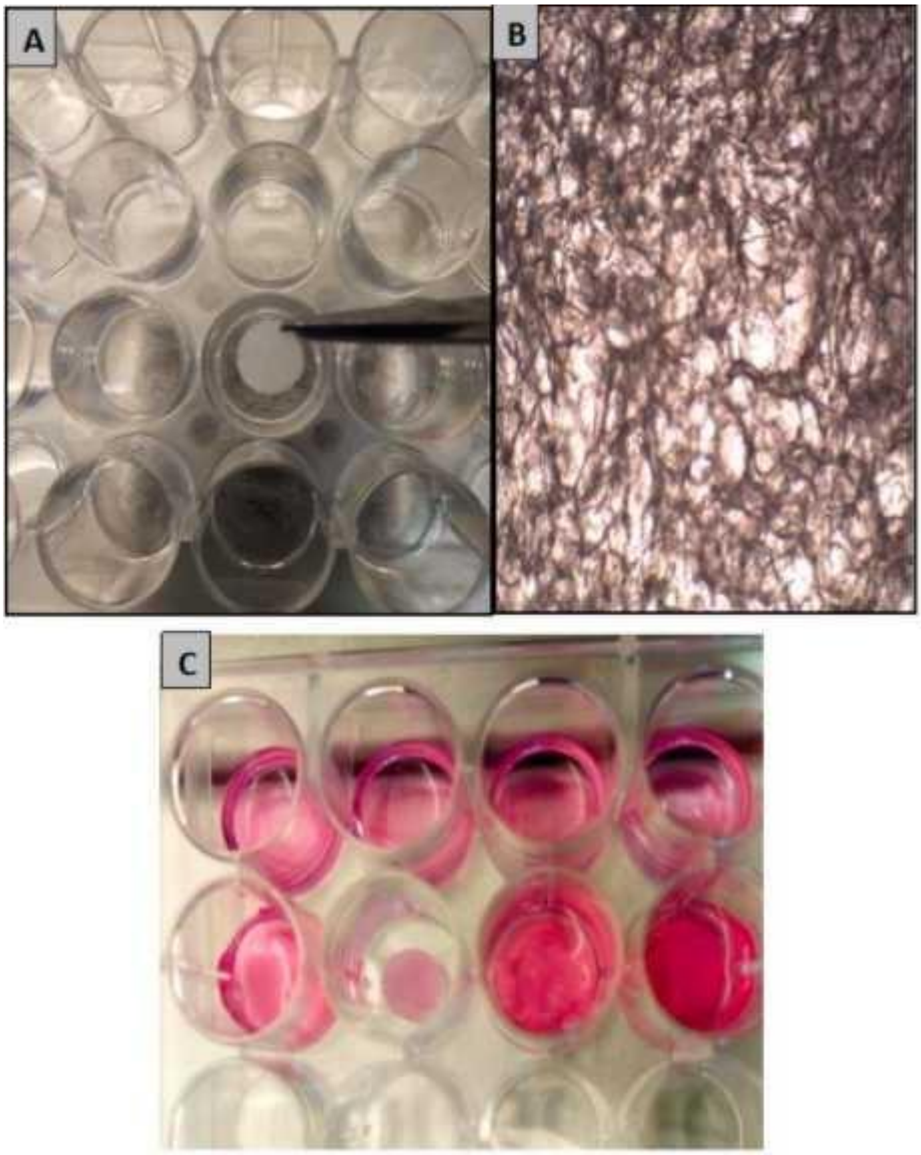

Fig. 1: cellusponge 3D scaffolds. A) Placing a cellusponge disk in the middle of each well in a 24- well plate B) Three-dimensional porous hydroxipropylcellulose scaffold under 10x magnification C) cellusponge positioning after adding 500µL of complete media into each well in a 24-well plate

Citation: Lara J Carroll, Brijesh Tiwari, James F Curtin, Janith Wanigasekara U-251MG Spheroid generation using a scaffold basedmethod protocol https://dx.doi.org/10.17504/protocols.io.bszqnf5w

This is an open access protocol distributed under the terms of the Creative Commons Attribution License (https://creativecommons.org/licenses/by/4.0/), which permits unrestricted use, distribution, and reproduction in any medium,

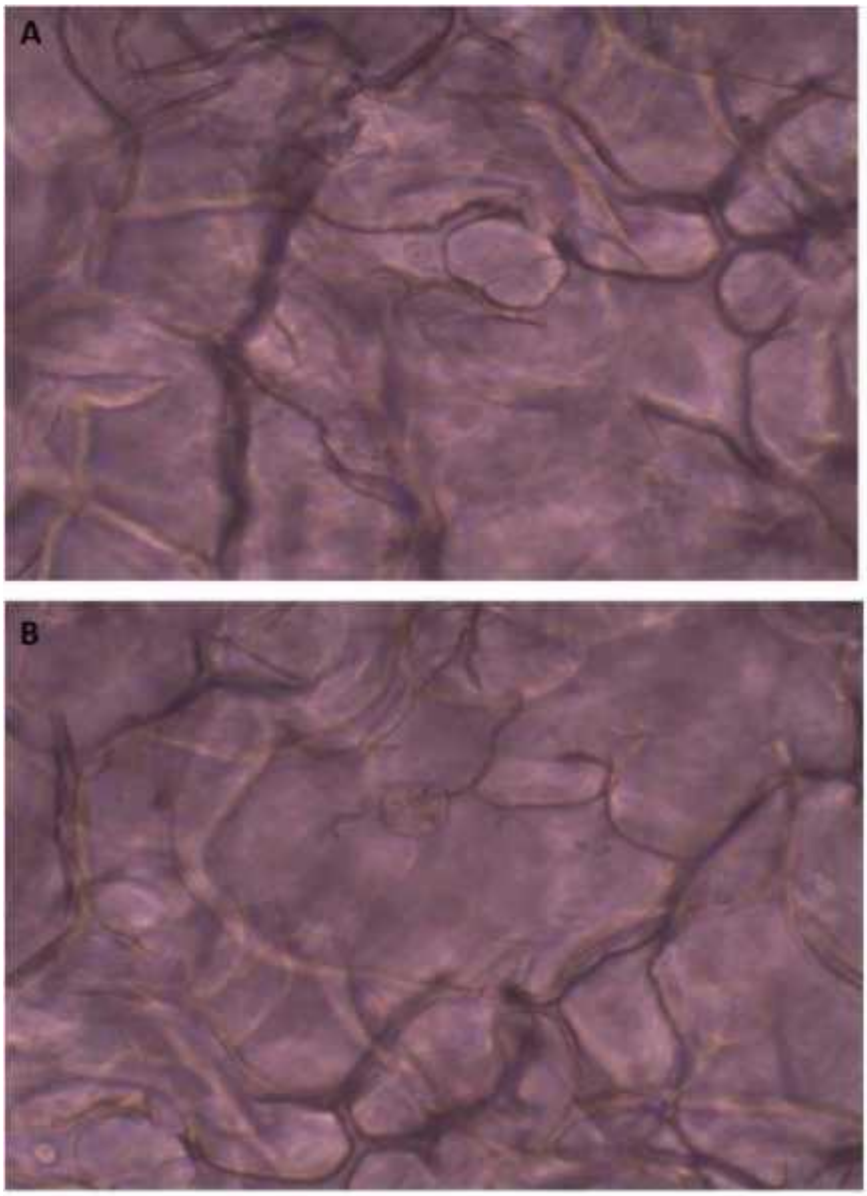

Citation: Lara J Carroll, Brijesh Tiwari, James F Curtin, Janith Wanigasekara U-251MG Spheroid generation using a scaffold basedmethod protocol https://dx.doi.org/10.17504/protocols.io.bszqnf5w

This is an open access protocol distributed under the terms of the Creative Commons Attribution License (https://creativecommons.org/licenses/by/4.0/), which permits unrestricted use, distribution, and reproduction in any medium,

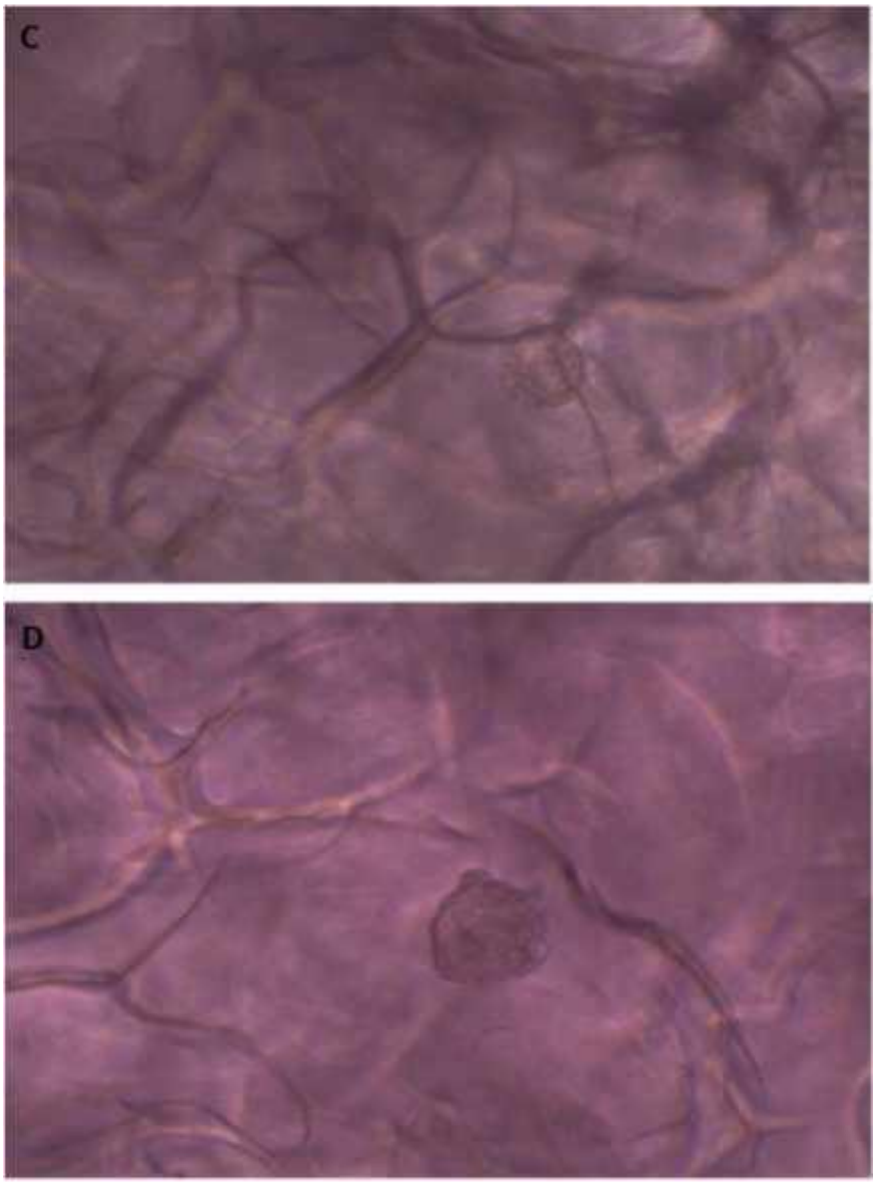

Fig. 2: U-251MG tumorspheroids formation in cellusponge 3D scaffolds. A) Tumorspheres formation after 1st day of incubation (Diameter - 22 µm), B) After 2nd day of incubation (Diameter - 49 µm), C) After 3rd day of incubation (Diameter - 70 µm), D) After 4th day of incubation (Diameter -110 µm).

Citation: Lara J Carroll, Brijesh Tiwari, James F Curtin, Janith Wanigasekara U-251MG Spheroid generation using a scaffold basedmethod protocol https://dx.doi.org/10.17504/protocols.io.bszqnf5w

This is an open access protocol distributed under the terms of the Creative Commons Attribution License (https://creativecommons.org/licenses/by/4.0/), which permits unrestricted use, distribution, and reproduction in any medium,

MATERIALS

- Complete medium (Dulbecco's Modified Eagle Medium + 10% V/V Fetal Bovine Serum + 1% Penicillin-Streptomycin (Sigma Aldrich)· Mammalian cells (U-251 MG human glioblastoma astrocytoma cells)
- Cellusponge 3D scaffolds (Sigma Alrich)
- Trypsin-EDTA solution(Sigma Aldrich)
- Phosphate Buffered Saline (PBS) 1X (pH41.7.4) [Sigma Aldrich]
- Trypan blue stain 0.4% (Thermo Fisher Scientific)
- Cell counter or cytometer
- Sterile reagent reservoirs
- Humidified incubator
- Multi-channel micropipette and tips
- 100-1000µl micropipette and tips
- Centrifuge with swinging bucket rotor
- Centrifuge tubes
- Bright-field microscope
- Cell culture flasks and dishes
- Serological pipettes

Laboratory coat and gloves must be worn at all times.

PROCEDURE

1. Once U-251MG cell flasks are 70-80% confluent, medium from the flask is aspirated and U- 251MG tumor cell monolayers re washed with 1X phosphate buffered saline (PBS; 5ml for a 25cm2 or 4ml for a 75cm2 flask), add 0.25 X Trypsin-EDTA cell dissociation enzyme (2ml for a 25cm2 or 4ml for a 75cm2 flask) and incubate cells at 37°C for 2-5 min

2. Check cell detachment under a microscope and neutralize cell dissociation enzyme with complete growth medium. The 0.25 X Trypsin-EDTA reagent is neutralized using 4 volumes of complete medium

3. Centrifuge U-251 MG single cell suspension at 5000rpm for 5 min, remove supernatant, tap the tube and re-suspend cell pellet in complete growth medium using a pipette. This should yield a single cell suspension without cell clusters

4. Cells with a viability >90% can be used for spheroid generation. Mix 10µl of cells with 10µl of Trypan Blue and count cells using a haemocytometer

5. For a 9mm scaffold, a cell suspension with the cell density of 5000k cells/ml must be obtained

6. Slowly place a cellusponge disk in the middle of each well in a 24-well plate

7. Added 100µL of U-251MG cell suspension on top of the sponge for a 9mm disk and incubated it for 3 hours for U-251MG cells. This will remove any air bubbles within the cellusponge

8. After incubation, 500µL of complete media was added to each well in a 24-well plate. Media should be added slowly along the edge of each well, not directly onto the cellusponge

9. The 24-well plate cellusponge was then incubated overnight in an incubator (37°C, 5% CO2, 95% humidity)

10. After overnight incubation, the seeded sponge is transferred into a new well plate, the media is replenished and the culture should be continued

Citation: Lara J Carroll, Brijesh Tiwari, James F Curtin, Janith Wanigasekara U-251MG Spheroid generation using a scaffold basedmethod protocol https://dx.doi.org/10.17504/protocols.io.bszqnf5w

This is an open access protocol distributed under the terms of the Creative Commons Attribution License (https://creativecommons.org/licenses/by/4.0/), which permits unrestricted use, distribution, and reproduction in any medium,

11. Tumor spheroid formation was observed within 3 days for U-251MG cell line

Citation: Lara J Carroll, Brijesh Tiwari, James F Curtin, Janith Wanigasekara U-251MG Spheroid generation using a scaffold basedmethod protocol https://dx.doi.org/10.17504/protocols.io.bszqnf5w

This is an open access protocol distributed under the terms of the Creative Commons Attribution License (https://creativecommons.org/licenses/by/4.0/), which permits unrestricted use, distribution, and reproduction in any medium,
