# Supplementary figures and images for: Three-Dimensional (3D) in vitro cell culture protocols to enhance glioblastoma research
Source: PLoS One. 2023 Feb 8;18(2):e0276248. doi: 10.1371/journal.pone.0276248 (PMC9907841; doi:10.1371/journal.pone.0276248)

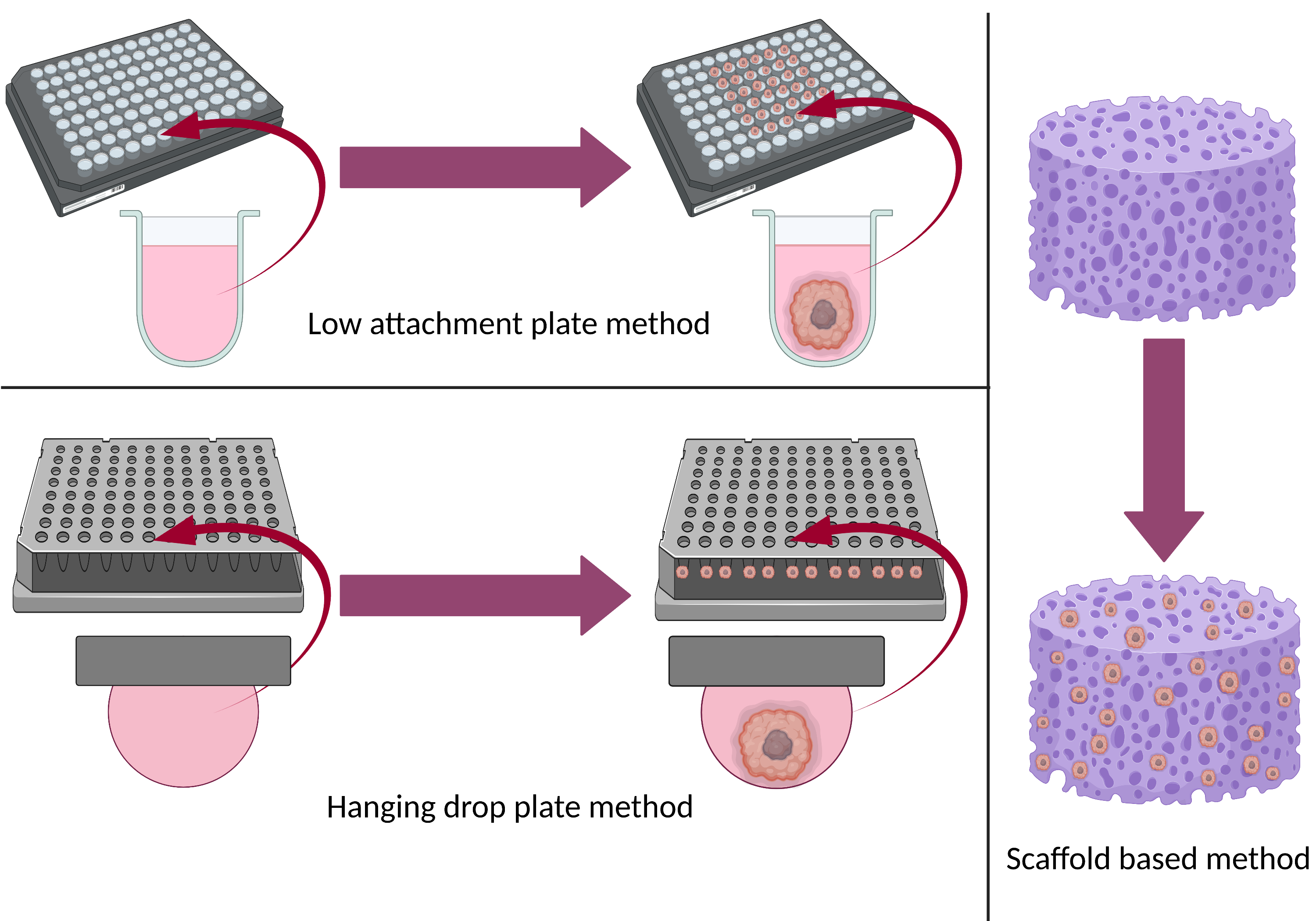

Supplement: S1 Graphical abstract — (PNG) [file pone.0276248.s004.png]
