# Supplementary material for: Three-Dimensional (3D) in vitro cell culture protocols to enhance glioblastoma research
Source: PLoS One. 2023 Feb 8;18(2):e0276248. doi: 10.1371/journal.pone.0276248 (PMC9907841; doi:10.1371/journal.pone.0276248)
Supplement: Attachment — Submitted filename: PONE-D-22-12260.pdf [file pone.0276248.s005.pdf]

PLOS ONE

Three-Dimensional (3D) in vitro cell culture protocols to enhance glioblastoma research

--Manuscript Draft--

**Manuscript Number:**	PONE-D-22-12260
**Article Type:**	Lab Protocol
**Full Title:**	Three-Dimensional (3D) in vitro cell culture protocols to enhance glioblastoma research
**Short Title:**	3D in vitro cell culture protocols
**Corresponding Author:**	James Curtin, PhD<br>Technological University Dublin<br>Dublin, Dublin IRELAND
**Keywords:**	3D Cell Culture; Glioblastoma; Hanging Drop Method; Low Adherence Method; Scaffold based method
**Abstract:**	3D cell culture models can help bridge the gap between in vitro cell cultures and in vivo responses by more accurately simulating the natural in vivo environment, shape, tissue stiffness, stressors, gradients and cellular response while avoiding the costs and ethical concerns associated with animal models. The inclusion of the third dimension in 3D cell culture influences the spatial organization of cell surface receptors that interact with other cells and imposes physical restrictions on cells in compared to 2D cell cultures. Spheroids' distinctive cyto-architecture mimics in vivo cellular structure, gene expression, metabolism, proliferation, oxygenation, nutrition absorption, waste excretion, and drug uptake while preserving cell–ECM connections and communication, hence influencing molecular processes and cellular phenotypes. This protocol describes the in vitro generation of tumourspheroids using the low attachment plate, hanging drop plate, and cellusponge natural scaffold based methods. The expected results from these protocols confirmed the ability of all these methods to create uniform tumourspheres.
**Order of Authors:**	Janith Wanigasekara
	Lara J Carroll
	Brijesh Tiwari
	Patrick J Cullen
	James Curtin, PhD
**Additional Information:**	
**Question**	**Response**
**Financial Disclosure**<br><br>Enter a financial disclosure statement that describes the sources of funding for the work included in this submission. Review the submission guidelines for detailed requirements. View published research articles from *PLOS ONE* for specific examples.<br><br>This statement is required for submission and **will appear in the published article** if the submission is accepted. Please make sure it is accurate.	Science Foundation Ireland (SFI) Grant Number 17/CDA/4653 (BT, PJC, JC)<br>Teagasc Walsh Fellowship 2017228 (JMW)

Powered by Editorial Manager® and ProduXion Manager® from Aries Systems Corporation

**Unfunded studies**<br>Enter: *The author(s) received no specific funding for this work.*<br><br>**Funded studies**<br>Enter a statement with the following details:<br>• Initials of the authors who received each award<br>• Grant numbers awarded to each author<br>• The full name of each funder<br>• URL of each funder website<br>• Have or will the sponsors or funders play any role in the study design, data collection (when applicable) and analysis, decision to publish, or preparation of the manuscript?<br>• **NO** - Include this sentence at the end of your statement: *The funders had and will not have a role in study design, data collection and analysis, decision to publish, or preparation of the manuscript.*<br>• **YES** - Specify the role(s) played.<br><br>* typeset	
**Competing Interests**<br><br>Use the instructions below to enter a competing interest statement for this submission. On behalf of all authors, disclose any competing interests that could be perceived to bias this work—acknowledging all financial support and any other relevant financial or non-financial competing interests.<br><br>This statement is required for submission and **will appear in the published article** if the submission is accepted. Please make sure it is accurate and that any funding sources listed in your Funding Information later in the submission form are also declared in your Financial Disclosure statement.<br><br>View published research articles from *PLOS ONE* for specific examples.	The authors have declared that no competing interests exist.

**NO authors have competing interests**<br><br>Enter: *The authors have declared that no competing interests exist.*<br><br>**Authors with competing interests**<br><br>Enter competing interest details beginning with this statement:<br><br>*I have read the journal's policy and the authors of this manuscript have the following competing interests: [insert competing interests here]*<br><br>* typeset	
**Ethics Statement**<br><br>Enter an ethics statement for this submission. This statement is required if the study involved:<br><br>• Human participants<br>• Human specimens or tissue<br>• Vertebrate animals or cephalopods<br>• Vertebrate embryos or tissues<br>• Field research<br><br>Write "N/A" if the submission does not require an ethics statement.<br><br>General guidance is provided below. Consult the submission guidelines for detailed instructions. **Make sure that all information entered here is included in the Methods section of the manuscript.**	This study used a human cell line (U-251 MG) obtained from a commercial cell bank. Ethics approval for the research was provided by the TU Dublin Ethics committee.

Powered by Editorial Manager® and ProduXion Manager® from Aries Systems Corporation

**Format for specific study types**<br><br>**Human Subject Research (involving human participants and/or tissue)**<br>• Give the name of the institutional review board or ethics committee that approved the study<br>• Include the approval number and/or a statement indicating approval of this research<br>• Indicate the form of consent obtained (written/oral) or the reason that consent was not obtained (e.g. the data were analyzed anonymously)<br><br>**Animal Research (involving vertebrate animals, embryos or tissues)**<br>• Provide the name of the Institutional Animal Care and Use Committee (IACUC) or other relevant ethics board that reviewed the study protocol, and indicate whether they approved this research or granted a formal waiver of ethical approval<br>• Include an approval number if one was obtained<br>• If the study involved *non-human primates*, add *additional details* about animal welfare and steps taken to ameliorate suffering<br>• If anesthesia, euthanasia, or any kind of animal sacrifice is part of the study, include briefly which substances and/or methods were applied<br><br>**Field Research**<br><br>Include the following details if this study involves the collection of plant, animal, or other materials from a natural setting:<br>• Field permit number<br>• Name of the institution or relevant body that granted permission	
**Data Availability**<br><br>Authors are required to make the data underlying their research findings fully available, without restriction.<br><br>If the manuscript reports sample data generated using the protocol, the data underlying those results must be made available at the time of publication. A Data	Yes - sample data are fully available without restriction

Powered by Editorial Manager® and ProduXion Manager® from Aries Systems Corporation

Availability Statement describing where the data can be found is required at submission. When legal or ethical requirements prohibit public data sharing, authors must indicate how researchers may obtain access to the data. See the PLOS Data Policy and FAQ for detailed information.<br><br>A Data Availability Statement describing where the data can be found is required at submission. Your answers to this question constitute the Data Availability Statement and **will be published in the article**, if accepted.<br><br>**Important:** Stating 'data available on request from the author' is not sufficient. If your data are only available upon request, select 'No' for the first question and provide justification in the text box.<br><br>For protocols without sample data or results, you must state this in your Data Availability Statement.<br><br>Do the authors confirm that EITHER the protocol does not report results OR that sample data will be made available?	
**Describe where the data may be found in full sentences. If you are copying our sample text, replace any instances of XXX with the appropriate details.**<br><br>• If the data are **held or will be held in a public repository**, include URLs, accession numbers or DOIs. If this information will only be available after acceptance, indicate this by ticking the box below. For example: *All XXX files are available from the XXX database (accession number(s) XXX, XXX.*).<br>• If the data are all contained **within the manuscript and/or Supporting Information files**, enter the following: *All relevant data are within the*	The data underlying the results presented in the study are available from the corresponding authors.

manuscript and its Supporting Information files.<br>• If neither of these applies but you are able to provide **details of access elsewhere**, with or without limitations, please do so. For example:<br><br>*Data cannot be shared publicly because of [XXX]. Data are available from the XXX Institutional Data Access / Ethics Committee (contact via XXX) for researchers who meet the criteria for access to confidential data.*<br><br>*The data underlying the results presented in the study are available from (include the name of the third party and contact information or URL).*<br>• This text is appropriate if the data are owned by a third party and authors do not have permission to share the data.<br><br>* typeset	
Additional data availability information:	

Powered by Editorial Manager® and ProduXion Manager® from Aries Systems Corporation

Manuscript

Click here to access/download;Manuscript;Wanigasekara 2022 PLOS One Manuscript.docx

LAB PROTOCOL

Three-Dimensional (3D) in vitro cell culture protocols to enhance glioblastoma research

Janith Wanigasekara[1,2,3,4]*, Lara J Carroll[1], Patrick J. Cullen[1,5], Brijesh Tiwari[3], James F. Curtin[1,2,4]*

[1] BioPlasma Research Group, School of Food Science and Environmental Health, Technological University Dublin, Dublin, Ireland.

[2] Environmental Sustainability & Health Institute (ESHI), Technological University Dublin, Dublin, Ireland.

[3] Department of Food Biosciences, Teagasc Food Research Centre, Ashtown, Dublin, Ireland.

[4] FOCAS Research Institute, Technological University Dublin, Dublin, Ireland.

[5] University of Sydney, School of Chemical and Biomolecular Engineering, Sydney, Australia.

* Corresponding authors. *Email address:* james.curtin@tudublin.ie and janith.manoharawanigasekara@tudublin.ie

Abstract

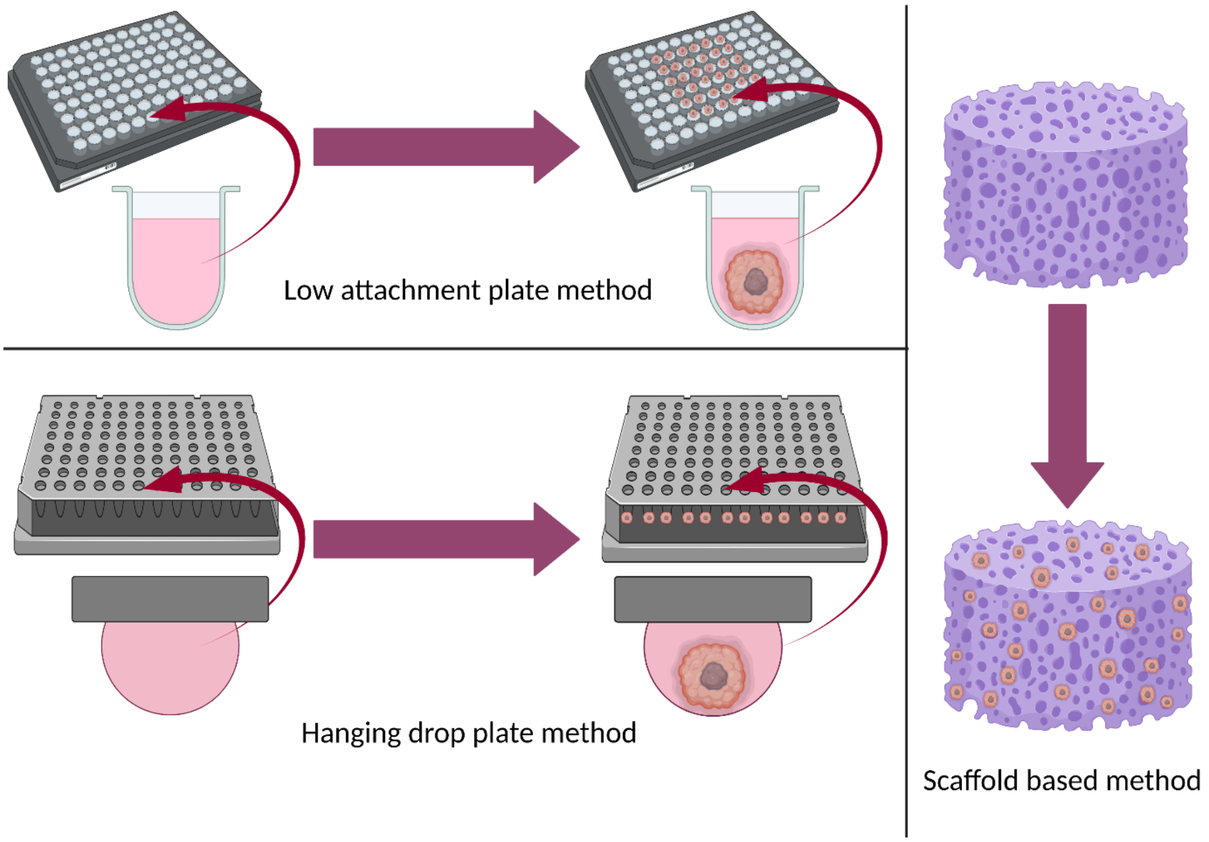

3D cell culture models can help bridge the gap between *in vitro* cell cultures and *in vivo* responses by more accurately simulating the natural *in vivo* environment, shape, tissue stiffness, stressors, gradients and cellular response while avoiding the costs and ethical concerns associated with animal models. The inclusion of the third dimension in 3D cell culture influences the spatial organization of cell surface receptors that interact with other cells and imposes physical restrictions on cells in compared to 2D cell cultures. Spheroids' distinctive cyto-architecture mimics *in vivo* cellular structure, gene expression, metabolism, proliferation, oxygenation, nutrition absorption, waste excretion, and drug uptake while preserving cell–ECM connections and communication, hence influencing molecular processes and cellular phenotypes. This protocol describes the *in vitro* generation of tumourspheroids using the low attachment plate, hanging drop plate, and cellusponge natural scaffold based methods. The expected results from these protocols confirmed the ability of all these methods to create uniform tumourspheres.

Introduction

Two-dimensional (2D) cell culture models have become a cornerstone of biological research due to its ease of use, cheap cost, and repeatability, however *in vivo* tissue complexity can only be reached utilizing Three-dimensional (3D) cell culture (1). 3D cell cultures are an improved *in vitro* cell culture technology that uses an artificially produced microenvironment to grow cells in three dimensions. Cells in 3D cell culture contain natural cell-cell interactions as well as cell–extracellular ma[illegible] component interactions, allowing them to proliferate *in vitro* in a microenvironment that closely reflects *in vivo* settings (2, 3). 3D cell culture is vital in drug testing, because it is capable of replacing both 2D cell culture and animal trials. The initial step of traditional drug development begins with 2D cell culture, followed by animal studies and clinical trials; around 95% of possible preclinical trials in all therapeutic areas fail to result in effective human treatments. The primary reason for this is that original data from 2D cell culture-based testing was deceptive and mispredicted cellular responses, resulting in enormous loss of time and resources and, eventually, delaying the identification of viable treatments (4, 5). 3D cell cultures are a simplified reductionist concept. When compared to a whole animal, it is very transparent and straightforward to mimic complicated processes like as growth, invasiveness, and toxicity (6). Thus the 3D cell culture technology can be utilize to enhance the quality of laboratory experiments and minimizing overall expenditure. It will also be able to develop cancer models, for preclinical screening and monitoring, as well as novel *in vivo* cancer therapeutic research (5, 7-9).

The main benefits of employing 3D cell culture for drug discovery include greater cell-cell contact, ECM-cell interactions, varied rates of cellular proliferation, oxygen and nutrition availability,

physiological gradients for nutrients, waste, signalling factors and drugs, and the additional influence of stroma, all of which assist in replicating natural tissue distribution (1, 5, 10). It can also simulate drug resistance, cellular microenvironment activity, and the expression of intrinsic and genetic variables (7, 10). The declared goal of the European REACH legislation is "To ensure a high level of protection of human health and the environment from the effects of hazardous chemicals. It strives for a balance: to increase our understanding of the possible hazards of chemicals, while at the same time avoiding unnecessary testing on animals" (European Chemicals Agency, 2020). 3D cell cultures complement the 3Rs principles of animal research (Replacement, Reduction, and Refinement) and REACH regulations by reducing the number of animals used in testing, as well as saving time, money, and ethical issues (1, 10). Animal testing is both costly and time consuming. Furthermore, if the animal is in pain or under stress throughout the experiment, it may alter the biochemical, physiological, and metabolic processes, which might misrepresent the efficacy and adverse effects of drugs (6, 9, 10). 3D spheroid cultures can be readily established in many cell culture facilities using anchorage-independent or anchorage-dependent methods. Three methods that can be established in most cell culture facilities are outlined in detail here, low attachment plate method, hanging drop method and scaffold based method.

To produce spheroids, anchorage independent/scaffold-free approaches rely on non-adherent cell-to-cell aggregation. This anchorage independent category includes low attachment plate and hanging drop plate technologies (11). Low attachment plates are culture plates with an ultra-low attachment hydrophilic polymer covering that promotes cell aggregation to create spheroids (2, 12). Cell adhesion to the culture surface is often mediated by ECM proteins such as collagen-I and fibronectin. The hydrophilic polymer covering prevents protein adsorption to the culture vessel surface, reducing monolayer cell adherence. Finally, low attachment plates stimulate cell aggregation via cell-cell and cell-ECM interactions, while limiting ECM interactions with the plastic surface (5). The benefits of employing low adhesion plates are straightforward, simple, efficient spheroid formation improved repeatability, reproducibility and handling, suitable for multicellular spheroids / co-culture and the ability to grow a wide range of tumour cell types (3, 13, 14). The disadvantage is that it is time consuming, lack of uniformity between spheroids, coated plates are expensive, continuous passage culture and toxicity analysis is difficult, that the success rate in long term passage is low, and not suitable for migration/invasion assays (4, 9, 15-17). When considering hanging drop plates are open bottomless wells that encourage the development of droplets of media that offer room for the creation of spheroids by self-aggregation via gravity and surface tension (5, 18). Because there is no surface to connect to, cells develop inside a bubble of growth medium, and spheroids dangle in open bottomless wells, which are frequently contained at the bottom of the plate to adjust the cells'

ambient humidity (12). The well can typically accommodate up to 50µl of media while recommended drop volume is 10-20 µl (15) and the spheroid size is determined by the cell density (2). This cell suspension droplet can be held in place by surface tension, and following 3D spheroid creation, it may be dispensed by adding an extra drop of media to the well and the spheroid loaded to ~~an~~ another normal well plate (9). The hanging drop plate method produce smaller size spheroids compared to low attachment plate method. The advantages of adopting the hanging drop plate method are the ability to create uniform size spheroids, the low cost, the ease of handling, the suitability for co-culturing, short term culture, and high throughput testing (3). The fundamental disadvantage of this approach is that medium changes are difficult, different drug treatments at different time periods are impossible, it is not ideal for long-term culture, and it has a small culture volume (9, 15).

Anchorage-dependent techniques employ pre-designed porous membranes and polymeric fabric meshes known as "scaffolds," which can be made of natural or synthetic materials (16). This physical support can give structures ranging from basic mechanical to extracellular matrix-like structures (1). 3D spheroids can be created by seeding cells into the 3D scaffold matrices or by distributing cells in a liquid matrix followed by solidification and polymerization. Cells are immersed in extracellular components and can begin cell-cell and cell-matrix interactions, as well as provide physical support for cell growth, adhesion, and proliferation (1). Fibronectin, collagen, laminin, gelatin, cellulose, chitosan, glycosaminoglycans, fibroin, agarose, alginate, starch, and human decellularized ECM are some of the natural scaffolds (14, 15, 19, 20). The benefits of employing biological scaffolds are that they are extremely comparable to *in vivo* settings, that they can manage similar composition/elasticity/porosity to achieve better ECM presentation, and that they can be combined with appropriate growth factors. It can also increase biocompatibility and reduce toxicity. Disadvantages include the fact that it is an expensive, time-consuming, complicated procedure that is not ideal for large-scale manufacturing, that it is difficult to separate cells from scaffold for further investigations such as flow cytometry and confocal imaging (15). Polymers such as polyglycolic acid, polylactic acid, polyorthoesters and aliphatic polyesters such as polycaprolactone (PCL), polystyrene (PS), polycaprolactone (PCL), polyethylene oxide (PEO), and polyethylene glycol (PEG) can be used to make synthetic scaffolds (1, 14, 15). The advantages of using synthetic scaffold include the ability to control porosity, stiffness, elasticity, and permeability, higher versatility, reproducibility, enhanced workability, ease of use, and mechanical qualities of synthetic materials that can be adjusted according to the cell culture required, and also their chemical composition is well characterized (11, 15). The disadvantages include a lack of biodegradation, which may impair cellular function, inability to remove cells and perform cytotoxicity tests.

Ultimately, 3D models must have high-throughput application, easy and standardized culture protocols and analytic methodologies to get proper outcomes (1). In the present study, we used three different methods to construct ~~an~~ *in vitro* 3D glioblastoma and epidermoid tumourspheroid models to closely mimic the natural *in vivo* environment, shape, and cellular response. This is the first time that we are reporting all the three different approaches for successful U-251MG human glioblastoma astrocytoma tumoursphere development.

Materials and methods

The part of this protocols described in this peer-reviewed article is published on protocols.io, and is included for printing as S1, S2, S3 Files with this article.

Low attachment plate method - dx.doi.org/10.17504/protocols.io.bszmnf46

Hanging drop plate method - dx.doi.org/10.17504/protocols.io.btstnnen

Scaffold based method - dx.doi.org/10.17504/protocols.io.bszqnf5w

Image J analysis

Tumor spheroid formation was visually confirmed daily using an Optika XDS-2 trinocular inverse microscope equipped with a Camera ISH500, and their mean diameters were analysed using "ImageJ version 1.53.e" software (http://imagej.nih.gov/ij/). ImageJ is a free software that can be used for manually counting the cell numbers and calculating the cellular size (area/ diameter). The ImageJ program was calibrated (set scale) using an image obtained from the same microscope with a known scale before it was used to calculate the cell size (in diameter). Following the calibration, the pictures of the tumorspheres were opened in the program, and a line was drawn across the diameter to measure the tumorsphere's size. The diameters of the spheroids were measured at least three times to obtain the mean and standard deviation.

Growth Analysis at different incubations

U-251 MG tumourspheres growth were analysed during different incubations (ranging from 24 to 168h). Cells (Initial seeding density was 10000 cells/ml) were seeded in the above mentioned Nunclon™Sphera™96-well-low attachment plates. Fresh media were added every third day by replenishing old media in each well without disturbing the tumorspheroids. In Hanging drop plate method, 5000 cells/well were seeded in the HDP1096 Perfecta3D® 96-well Plate. While in scaffold based method, 5000k cells/ml were seeded in the hydroxipropylcellulose scaffold. The spheroid formation and growth were monitored daily by using an inverted phase-contrast microscope, and the sizes of the spheroids were measured as explained above for at least three independent experiments.

Growth analysis at different seeding densities

For growth analysis, varying numbers of U-251 MG cells (ranging from 2000 to 40 000 cells/ml) were seeded in the above mentioned Nunclon™ Sphera™ 96-well-low attachment plates for 96 hours. Fresh media were added every third day by replenishing old media in each well without disturbing the tumorspheroids. In Hanging drop plate method, U-251 MG cells (ranging from 1000 to 10000

cells/well) were seeded in the above mentioned HDP1096 Perfecta3D® 96-well Plate. While in scaffold based method, varying numbers of U-251 MG cells (ranging from $1x10^6$ to $6x10^6$ cells/ml) were seeded in the hydroxipropylcellulose scaffold. The spheroid formation was monitored after 96h by using an inverted phase-contrast microscope, and the sizes of the spheroids were measured as explained above for at least three independent experiments.

Spheroid cells health analysis

Spheroid cell health was analysed using Alamar Blue™ cell viability reagent (Thermo Fisher Scientific). After the post treatment incubation, tumourspheres were washed with sterile phosphate-buffered saline (PBS), and incubated for 3 h at 37°C with a 10% Alamar Blue™ solution (10). Fluorescence was measured using an excitation wavelength of 530 nm and an emission wavelength of 590 nm with a Varioskan Lux multi-plate reader (Thermo Scientific). The fluorescence signals were normalized by spheroid size (in diameter); a higher ratio indicates healthier spheroids. All experiments consisted of at least three independent tests.

<u>Expected results</u>

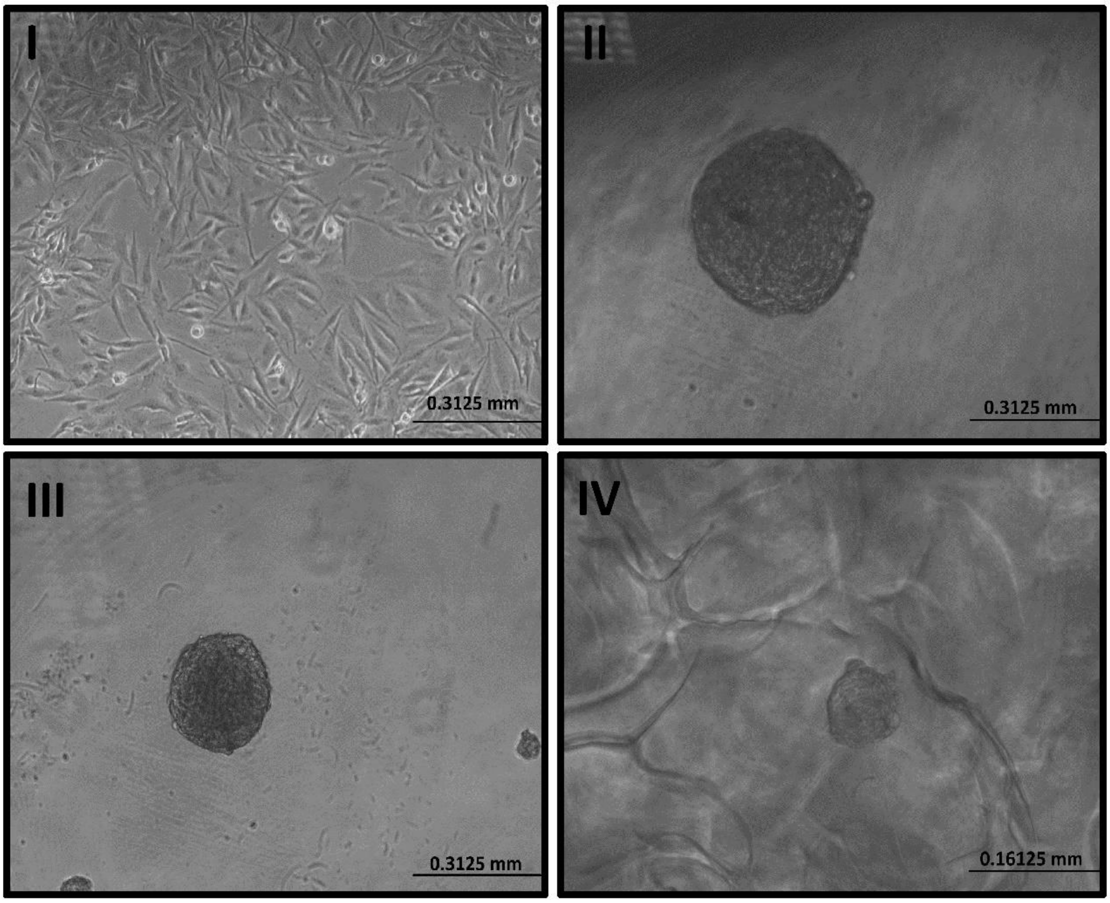

Figure 1 - Development of U-251MG human glioblastoma astrocytoma 3D *in vitro* cell culture model. I). Image of U-251MG 2D cells in T75 flask II) 3D tumoursphere constructed in low adhesion plate. III). 3D tumoursphere constructed in hanging drop plate. IV) 3D tumoursphere constructed in hydroxipropylcellulose scaffold. Tumour spheroid formation was visually confirmed using an Optika XDS-2 trinocular inverse microscope equipped with a Camera ISH500.

Three distinct approaches such as Low attachment plate (Figure 1-II), (S1), Hanging drop plate (Figure 1-III), (S2), and scaffold based methods (Figure 1-IV), (S3) were used to create 3D spheroids of U-251MG human glioblastoma astrocytoma. This facilitated 3D cell–cell and cell–ECM interactions and mirrored the diffusion-limited distribution of oxygen, nutrients, metabolites, and signaling molecules seen in the microenvironment of *in vivo* tumours. Most research to date has used 2D cell culture (Figure 1-I), which has limitations as experimental models to predict biological responses, as explained previously.

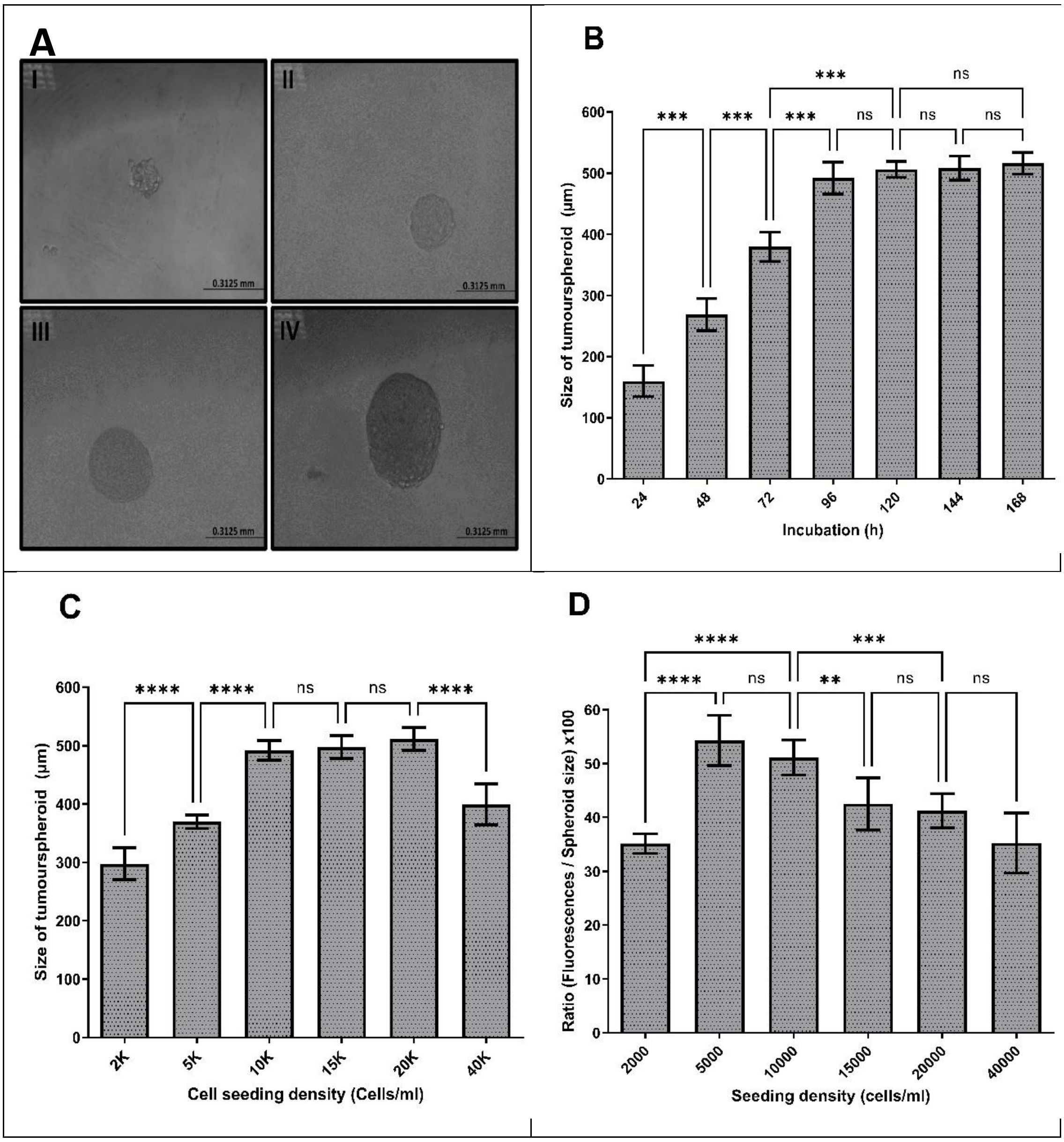

Figure 2: Development of U-251 MG human glioblastoma astrocytoma 3D *in vitro* cell culture models using low attachment plate method. A) U-251MG tumourspheroids formation, I) Tumourspheres formation after 24 h incubation, II) after 48 h incubation, III) after 72 h of incubation, IV) after 96h of incubation. B) U-251 MG tumoursphere growth (diameter in µm) analysis during different incubations. C) U-251 MG Growth analysis after 96h incubation (diameter in µm) at increasing seeding density. The mean of the diameter was used to plot the values on columns and analysed using one-way ANOVA with Tukey's post-test (ns, not significant ($p > 0.05$); *$p < 0.05$; **$p < 0.01$, ***$p < 0.001$; ****$p < 0.0001$). D) U-251 MG Spheroid cell health analysed and a higher ratio indicates healthier spheroids. The mean of the [(fluorescence / spheroid size) x 100] was used to plot the values on columns and analysed using one-way ANOVA with Tukey's post-test (ns, not significant ($p > 0.05$); *$p < 0.05$; **$p < 0.01$, ***$p < 0.001$; ****$p < 0.0001$).

The U-251MG human glioblastoma astrocytoma spheroid formation and growth were monitored daily by using an inverted phase-contrast microscope, and their mean diameters were analysed using "ImageJ version1.53.e" software for at least three independent experiments. U-251MG tumoursphere growth during low attachment plate method was found to be significantly increased with the incubation time, the size ranging from 135µm, 229 µm, 323 µm and 461 µm for 24 to 96 h incubation respectively (Figure 2A). The optimum U251 MG tumourspheroids formation was observed within 96h of incubation for the 10000 cells/ml initial seeding density. One-way analysis of variance (ANOVA) demonstrated that there is a significant difference in tumoursphere diameter during 24 -96h incubation, while, there was no significant difference during 96h to 168h incubation (Figure 2 B). It was also observed that exponential growth (Log) was achieved within the initial 4 days of growth, after which the growth curve became stationary.

For growth analysis, varying numbers of U-251 MG cells (ranging from 2000 to 40 000 cells/ml) were seeded in the Nunclon™ Sphera™ 96-well-low attachment plates as explained above. The largest U-251 MG tumourspheres were observed with 10 000, 15 000, and 20 000 cells/ml initial seeding densities after 96h incubation. One-way ANOVA demonstrated that there is a significant difference in tumoursphere diameter between each initial seeding densities as shown in Figure 1C. However, there was no significant difference between diameters in 10 000, 15 000, and 20 000 cells/ml seeding densities.

U251-MG cell health analysed after 96h incubation using Alamar Blue™ cell viability reagent as explained above and the fluorescence signals were normalized by spheroid size (diameter in µm). A higher ratio suggests that the spheroids are healthier. During U251-MG growth confirmed that 5000 and 10 000 cells/ml initial seeding densities were having highest spheroids cell health. One-way

ANOVA confirmed that there was no significant difference in tumoursphere health during 5000 and 10 000 cells/ml.

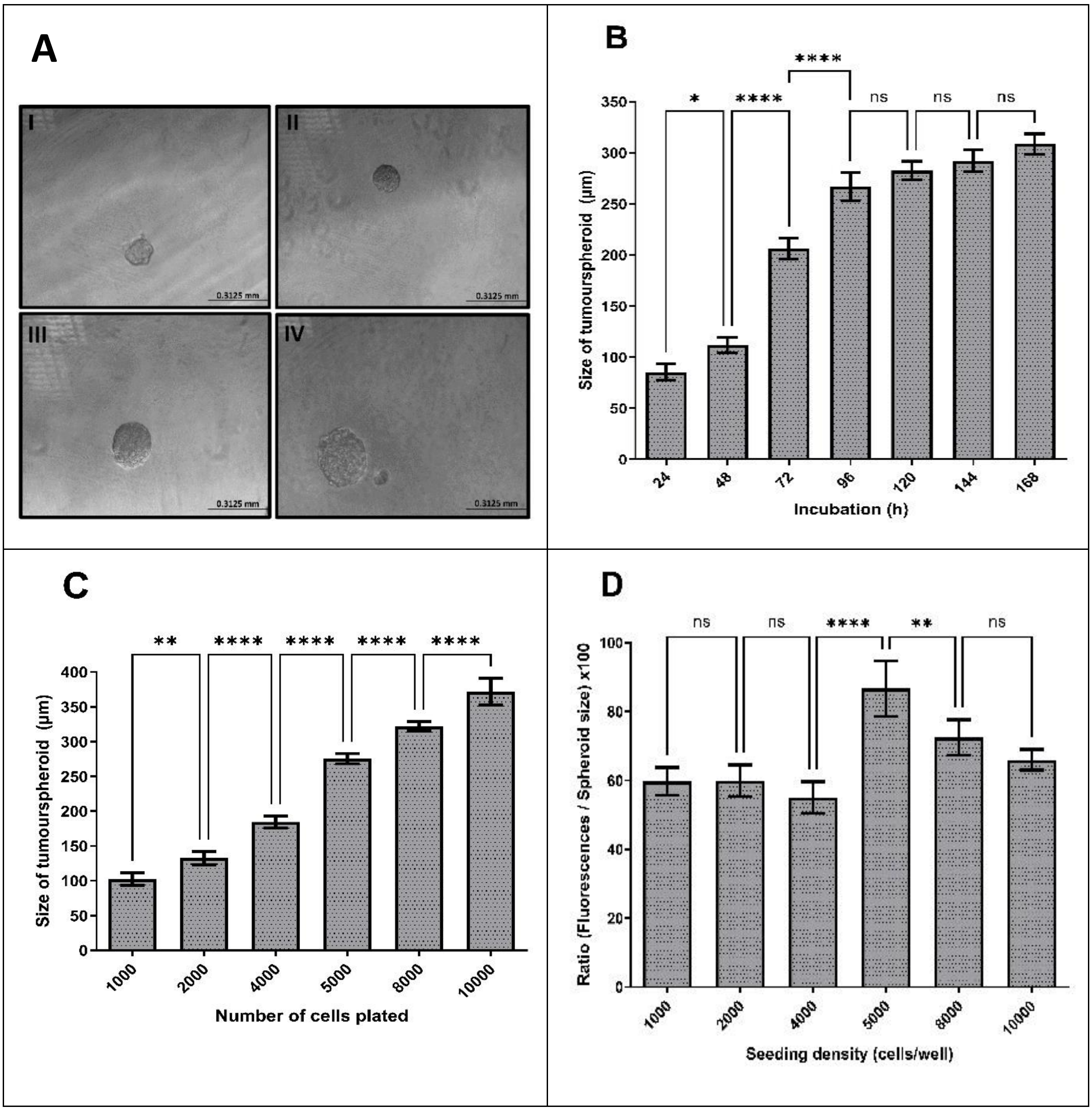

Figure 3: Development of U-251 MG human glioblastoma astrocytoma 3D *in vitro* cell culture model using hanging drop plate method. A) U-251MG tumourspheroids formation, I) Tumourspheres formation after 24 h incubation, II) after 48 h incubation, III) after 72 h of incubation, IV) after 96h of incubation. B) Tumoursphere growth (diameter in µm) analysis during different incubations. C) Growth analysis after 96h incubation (diameter in µm) at increasing seeding density. The mean of the diameter was used to plot the values on columns and analysed using one-way ANOVA with Tukey's post-test (ns, not significant ($p > 0.05$); $*p < 0.05$; $**p < 0.01$, $***p < 0.001$; $****p < 0.0001$). D) Spheroid cell health analysed and a higher ratio indicates healthier spheroids. The mean of the [(fluorescence / spheroid size) x 100] was used to plot the values on columns and analysed using

one-way ANOVA with Tukey's post-test (ns, not significant ($p > 0.05$); *$p < 0.05$; **$p < 0.01$, ***$p < 0.001$; ****$p < 0.0001$).

U-251MG tumoursphere growth during hanging drop plate method was shown to be considerably enhanced with the incubation time, with size ranging from 105µm, 139 µm, 208 µm and 269 µm for 24 to 96 h incubation respectively (Figure 3A). The optimum tumourspheroids formation attained after 96h incubation by achieving a size range 251-285 µm for the 5000 cells/well initial seeding density. One-way ANOVA indicated that there is a significant difference in tumoursphere diameter during 48 - 96h incubation, while, there was no significant difference during 96h to 168h incubation (Figure 3B).

For growth analysis, varying numbers of U-251 MG cells (ranging from 1000 to 10 000 cells/well) were seeded in the HDP1096 Perfecta3D® 96-Well Hanging Drop Plates and the mean sizes were computed after 96h of incubation. The largest U-251 MG tumourspheres were observed with 10 000 cells/well initial seeding densities after 96h incubation. As illustrated in Figure 3C, one-way ANOVA revealed a significant difference in tumoursphere diameter between each initial seeding density.

During U251-MG spheroids cell health investigation, it was established that the initial seeding density of 5000 cells/well had the best spheroids cell health. The substantial difference in 4000 to 5000 cells/well and 5000 to 8000 cells/well was verified by one-way ANOVA, however there was no significant difference in tumoursphere health at the other seeding densities (Figure 3D).

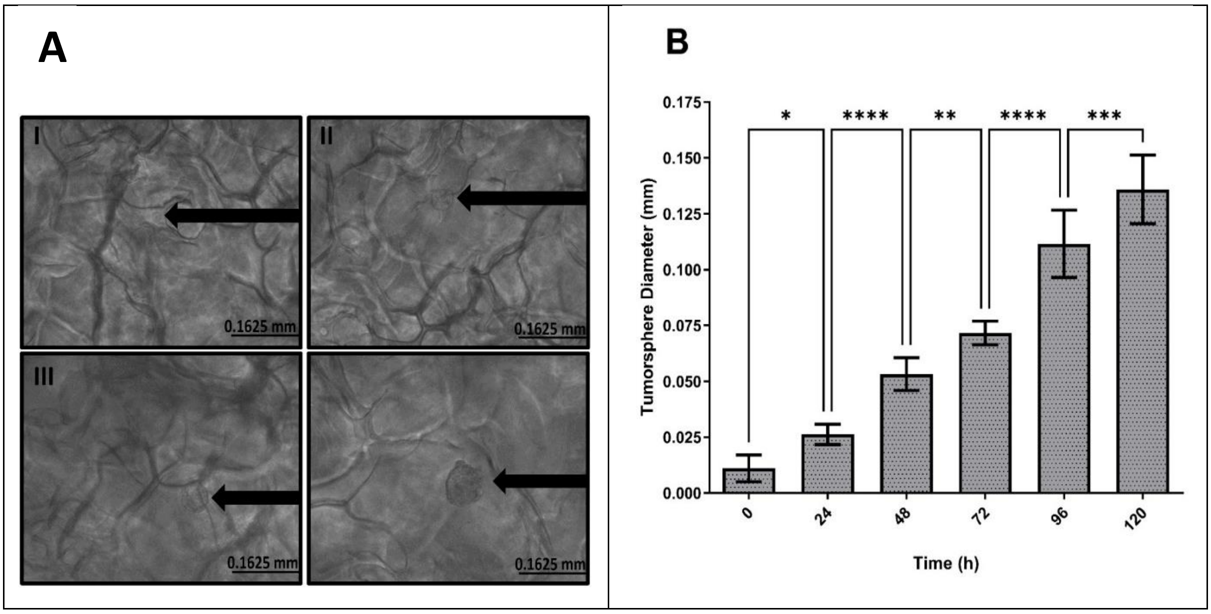

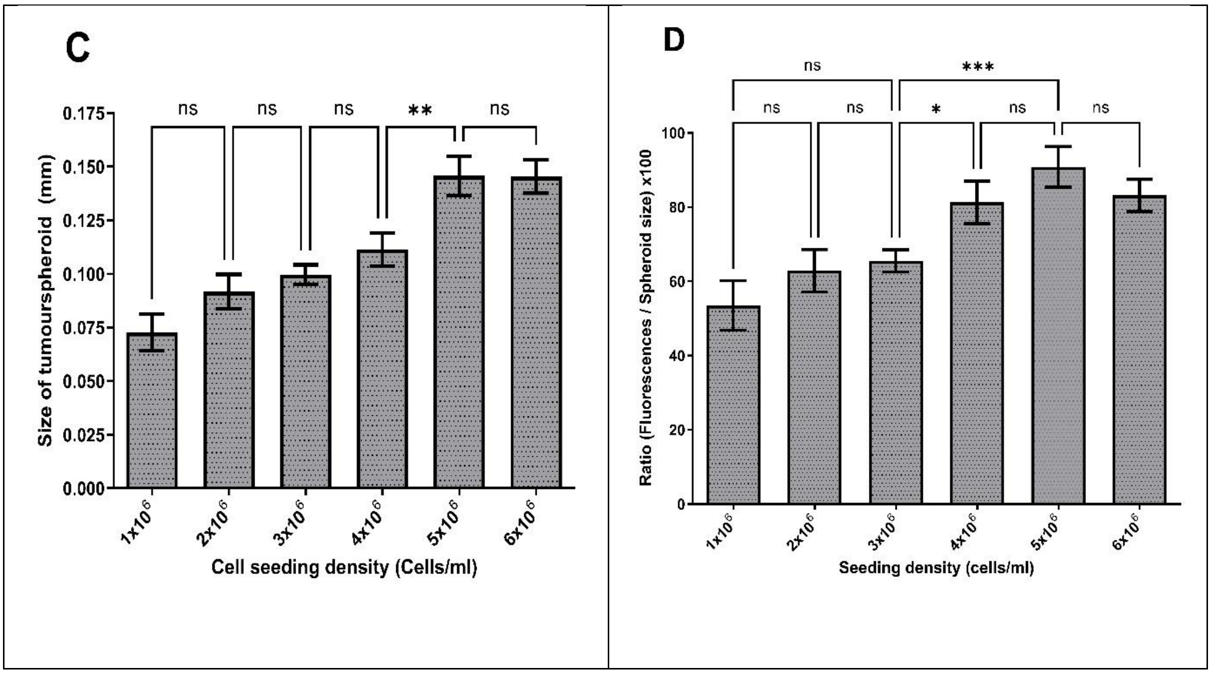

Figure 4: Development of U-251 MG human glioblastoma astrocytoma 3D *in vitro* cell culture model using Cellusponge 3D scaffolds. A) U-251MG tumourspheroids formation, I) Tumourspheres formation after 24 h incubation, II) after 48 h incubation, III) after 72 h of incubation, IV) after 96h of incubation. B) Tumoursphere growth (diameter in mm) analysis during different incubations and all the data points were statistically significant. C) U-251 MG Growth analysis after 120h incubation (diameter in mm) at increasing seeding density. The mean of the diameter was used to plot the values on columns and analysed using one-way ANOVA with Tukey's post-test. (ns, not significant ($p > 0.05$); *$p < 0.05$; **$p < 0.01$, ***$p < 0.001$; ****$p < 0.0001$). D) U-251 MG Spheroid cell health analysed and a higher ratio indicates healthier spheroids. The mean of the [(fluorescence / spheroid size) x 100] was used to plot the values on columns and analysed using one-way ANOVA with Tukey's post-test (ns, not significant ($p > 0.05$); *$p < 0.05$; **$p < 0.01$, ***$p < 0.001$; ****$p < 0.0001$).

U-251MG tumoursphere growth in hydroxipropylcellulose 3D scaffold was shown to be considerably enhanced with incubation time, with sizes ranging from 22µm, 49µm, 70µm, and 110 µm for 24 to 96 h incubation, respectively (Figure 4A). The largest tumourspheroids formation attained after 120h incubation by achieving a size range 0.110-0.156 mm for the 5000k cells/ml initial seeding density. One-way ANOVA indicated that there is a significant difference in tumoursphere diameter during throughout the incubation (Figure 4B).

For growth analysis, varying numbers of U-251 MG (ranging from $1x10^6$ to $6x10^6$ cells/ml) were seeded in the hydroxipropylcellulose 3D scaffolds. Fresh media were added every third day by replenishing old media in each well without disturbing the scaffolds and the mean sizes were calculated after 120h of incubation. The largest tumourspheres were detected with $5x10^6$ and $6x10^6$ cells/ml initial seeding

densities after 120h incubation. One-way ANOVA verified that there is a significant difference in tumoursphere diameter between $4x10^6$ and $5x10^6$ seeding densities as shown in Figure 4C.

U251-MG spheroids cell health analysed after 120h incubation as explained above, confirmed that $5x10^6$ cells/ml initial seeding density was having highest spheroids cell health. One-way ANOVA confirmed that there was no significant difference in tumoursphere health during $4x10^6$, $5x10^6$ and $6x10^6$ cells/ml. While there was a significant difference between $3x10^6$ and $4x10^6$ densities as shown in Figure 4D.

Supporting information

S1 File. U-251MG Spheroid generation using low attachment plate method protocol
Also available on protocols.io.
https://www.protocols.io/view/u-251mg-spheroid-generation-using-low-attachment-p-bszmnf46.pdf

S2 File. U-251MG Spheroid Generation Using Hanging Drop Method Protocol
Also available on protocols.io.
https://www.protocols.io/view/u-251mg-spheroid-generation-using-hanging-drop-met-btstnnen.pdf

S3 File. U-251MG Spheroid generation using a scaffold based method protocol
Also available on protocols.io.
https://www.protocols.io/view/u-251mg-spheroid-generation-using-a-scaffold-based-bszqnf5w.pdf

Acknowledgments

This study was supported by Science Foundation Ireland (SFI) under Grant Number 17/CDA/4653 and funded through Teagasc Walsh Fellowship. The authors also thank TU Dublin, ESHI, and FOCAS Research Institutes for the use of facilities and support of technical staff.

Data availability statement

The data that support the findings of this study are available from the corresponding author upon reasonable request.

References

1. Paradiso F, Serpelloni S, Francis LW, Taraballi F. Mechanical Studies of the Third Dimension in Cancer: From 2D to 3D Model. International Journal of Molecular Sciences. 2021;22(18).

2. Foglietta F, Canaparo R, Muccioli G, Terreno E, Serpe L. Methodological aspects and pharmacological applications of three-dimensional cancer cell cultures and organoids. Life Sciences. 2020;254.
3. Reidy E, Leonard NA, Treacy O, Ryan AE. A 3D View of Colorectal Cancer Models in Predicting Therapeutic Responses and Resistance. Cancers. 2021;13(2).
4. Brancato V, Oliveira JM, Correlo VM, Reis RL, Kundu SC. Could 3D models of cancer enhance drug screening? Biomaterials. 2020;232.
5. Belfiore L, Aghaei B, Law AMK, Dobrowolski JC, Raftery LJ, Tjandra AD, et al. Generation and analysis of 3D cell culture models for drug discovery. European Journal of Pharmaceutical Sciences. 2021;163.
6. Pampaloni F, Reynaud EG, Stelzer EHK. The third dimension bridges the gap between cell culture and live tissue. Nature Reviews Molecular Cell Biology. 2007;8(10):839-45.
7. Farhat J, Pandey I, AlWahsh M. Transcending toward Advanced 3D-Cell Culture Modalities: A Review about an Emerging Paradigm in Translational Oncology. Cells. 2021;10(7).
8. Caragher S, Chalmers AJ, Gomez-Roman N. Glioblastoma's Next Top Model: Novel Culture Systems for Brain Cancer Radiotherapy Research. Cancers. 2019;11(1).
9. Jensen C, Teng Y. Is It Time to Start Transitioning From 2D to 3D Cell Culture? Frontiers in Molecular Biosciences. 2020;7.
10. Wanigasekara J, Barcia C, Cullen PJ, Tiwari B, Curtin JF. Plasma induced reactive oxygen species-dependent cytotoxicity in glioblastoma 3D tumourspheres. Plasma Processes and Polymers.
11. Sayde T, El Hamoui O, Alies B, Gaudin K, Lespes G, Battu S. Biomaterials for Three-Dimensional Cell Culture: From Applications in Oncology to Nanotechnology. Nanomaterials. 2021;11(2).
12. Mapanao AK, Voliani V. Three-dimensional tumor models: Promoting breakthroughs in nanotheranostics translational research. Applied Materials Today. 2020;19.
13. Fontana F, Raimondi M, Marzagalli M, Sommariva M, Gagliano N, Limonta P. Three-Dimensional Cell Cultures as an In Vitro Tool for Prostate Cancer Modeling and Drug Discovery. International Journal of Molecular Sciences. 2020;21(18).
14. Alzeeb G, Metges JP, Corcos L, Le Jossic-Corcos C. Three-Dimensional Culture Systems in Gastric Cancer Research. Cancers. 2020;12(10).
15. Lv DL, Hu ZT, Lu L, Lu HS, Xu XL. Three-dimensional cell culture: A powerful tool in tumor research and drug discovery. Oncology Letters. 2017;14(6):6999-7010.
16. Stankovic T, Randelovic T, Dragoj M, Buric SS, Fernandez L, Ochoa I, et al. In vitro biomimetic models for glioblastoma-a promising tool for drug response studies. Drug Resistance Updates. 2021;55.
17. Park Y, Huh KM, Kang SW. Applications of Biomaterials in 3D Cell Culture and Contributions of 3D Cell Culture to Drug Development and Basic Biomedical Research. International Journal of Molecular Sciences. 2021;22(5).
18. Dundar B, Markwell SM, Sharma NV, Olson CL, Mukherjee S, Brat DJ. Methods for in vitro modeling of glioma invasion: Choosing tools to meet the need. Glia. 2020;68(11):2173-91.
19. Tomas-Bort E, Kieler M, Sharma S, Candido JB, Loessner D. 3D approaches to model the tumor microenvironment of pancreatic cancer. Theranostics. 2020;10(11):5074-89.
20. Fisher MF, Rao SS. Three-dimensional culture models to study drug resistance in breast cancer. Biotechnology and Bioengineering. 2020;117(7):2262-78.

Graphical Abstract

Click here to access/download;Figure;Graphical Abstract.png

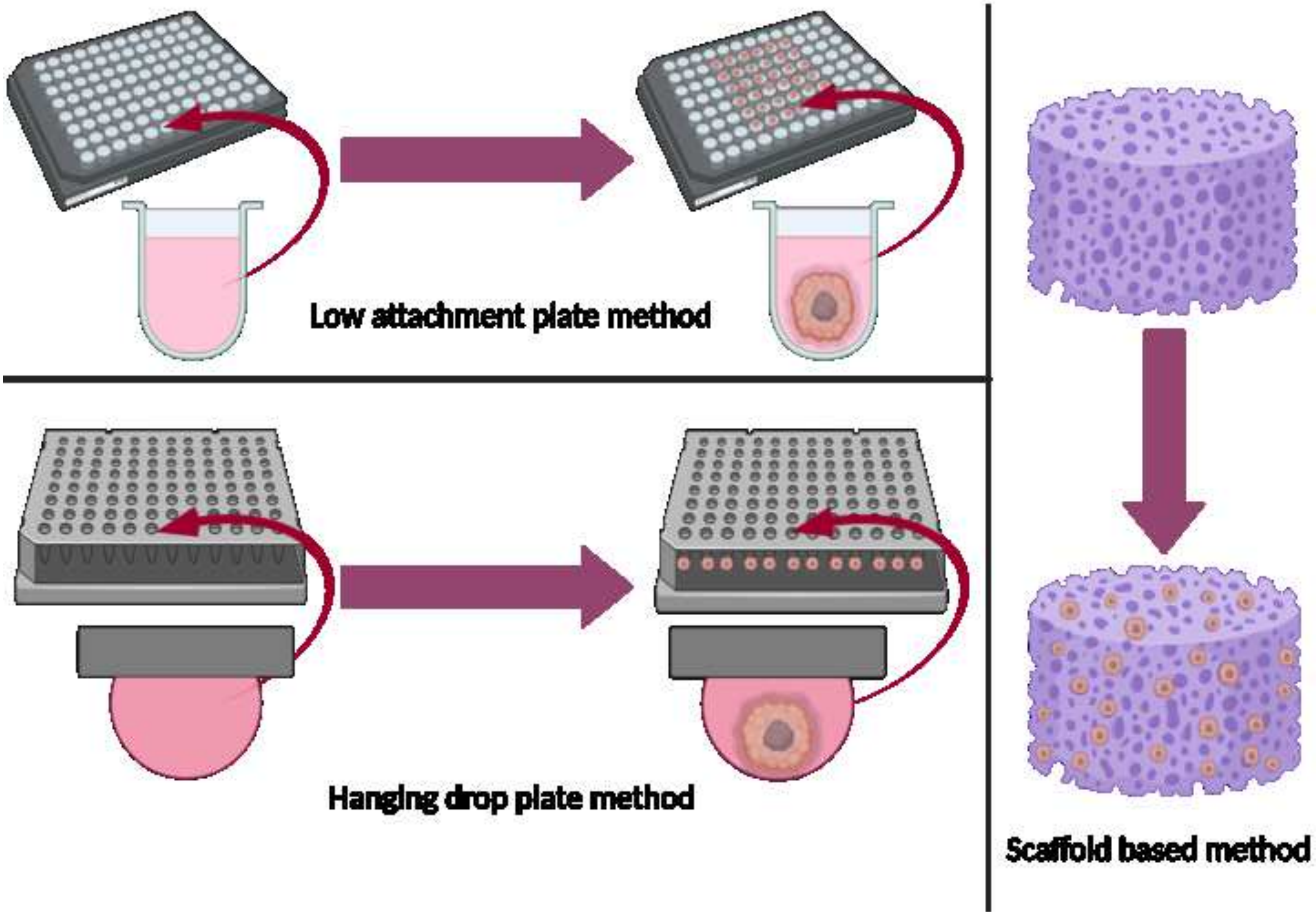

Click here to access/download
Supporting Information
S1.pdf

Click here to access/download
Supporting Information
S2.pdf

Supporting Information S3

Click here to access/download
Supporting Information
S3.pdf
