# Supplementary material for: Three-Dimensional (3D) in vitro cell culture protocols to enhance glioblastoma research
Source: PLoS One. 2023 Feb 8;18(2):e0276248. doi: 10.1371/journal.pone.0276248 (PMC9907841; doi:10.1371/journal.pone.0276248)
Supplement: Attachment — Submitted filename: Response to Reviewers.docx [file pone.0276248.s006.docx]

**Three-Dimensional (3D) in vitro cell culture protocols to enhance glioblastoma research**

**Reviewer #1**: Review for PLOS One – PONE-D-22-12260
Wanigasekara et al. “Three-Dimensional (3D) in vitro cell culture protocols to enhance glioblastoma research.”

The manuscript by Wanigasekara et al. with the title “Three-Dimensional (3D) in vitro cell culture protocols to enhance glioblastoma research.” describes the generation of glioblastoma tumorspheroids. Three different approaches are presented, based on the utilization of low attachment plates, hanging drop plates, and cellusponge natural scaffold. Using different cell numbers and incubation times, the authors investigate the growth of these glioblastoma tumorspheroids associated with the three different experimental approaches.

In general, the presented topic touches on an important aspect, as there is a lack of preclinical models recapitulating glioblastoma biology and the associated tumor microenvironment. However, the manuscript is neither comprehensive enough nor sufficiently detailed to support other researchers in the field with the advancement of glioblastoma 3D culture and translational research.

Major concerns:

1. In line with the existing knowledge on molecular heterogeneity of glioblastoma, the investigation of only one cell line is a major drawback of the presented manuscript.

Thank you for the suggestion. We repeated all the experiments with two more glioblastoma cell lines, and now in this current version we are presenting data for U-251MG, U-87 MG and A172 human glioblastoma cell lines. Our protocols were confirmed effective using three glioblastoma cell lines and can be applied to each cell line without changing any parameters.

2. The methodological section is partly presented online, however it remains unclear how data were quantified. For example, while at least three experiments were performed, it is not known how many spheroids were measured within each of the three biological repetitions.

An extended version of the methodology is included in the current version of the manuscript. Lines 140 to 205 and 248 to 272 show updated methodology. Also, more information about three biological repetitions was added in the lines from 223-225, 233-236, and 245-247.

3. The manuscript does do not offer new perspectives into how the described 3D models might be of relevance to glioblastoma research. For example: Are these assays adaptable to further functional endpoints? Does the Cellulsponge scaffold mimic physiological architecture of the brain or glioblastoma tumor? etc.

Thank you. The importance of 3D models in glioblastoma research is discussed further in the updated discussion section, which can be seen in lines 274-280, 319-321, 375-385, 429-435, 480-489, and 515-533.

4. The manuscript lacks a critical discussion of the current literature on the topic of glioblastoma 3D culture as well as specific and relevant conclusions on how to move forward in this research field.

Updated discussion can be seen from the lines 274-280, 319-321, 375-385, 429-435, 480-489, and 515-533.

**Reviewer #2**: The manuscript “Three-Dimensional (3D) in vitro cell culture protocols to enhance glioblastoma research” provide a good protocol for different 3D cultures of GBM.
I only miss a discussion, where the author reflects on the data presented from the 3 models. I think a discussion would complement the introduction part and the results.

Thank you for the suggestion. We Updated our discussion, can be seen from the lines 274-280, 319-321, 375-385, 429-435, 480-489, and 515-533.

**Reviewer #3**: The manuscript by Wanigasekara et al. entitled „Three-Dimensional (3D) in vitro cell culture protocols to enhance glioblastoma research“ describes different ways to generate tumor spheroids from cultured astrocytoma cell lines. This protocol is interesting and helpful per se, however, it is not acceptable in ist current form.

Major points:

1) The protocols were established on one cell line: U-251. This cell line is long-term cultured and used in high passage. As a result, a genetic drift can be anticipated, accompanied by variations in phenotypic marker expression and an increased growth rate in vitro. Particularly, this was previously reported for U-251 cells (Torsvik et al., Cancer Med. 2014 Aug;3(4):812-24). Hence, experiments must be repeated with primary cells freshly taken from glioblastoma patients.

Thank you for the suggestion. We repeated all the experiments with two other widely used human glioblastoma cell lines, and now in this current version we are presenting data for U-251MG, U-87 MG and A172 cells. Our protocols were confirmed effective using three glioblastoma cell lines and can be applied to each cell line without changing any parameters.

2) The protocol lacks any functional analyses, such as drug response testing (at least towards the most commonly used drug TMZ) or gene expression studies (e.g. changes in expression profiles between individual methods for tumorspheroid formation).

We completed a functional assay using TMZ and the cytotoxicity was analysed for U-251MG, U-87 MG, and A172 human glioblastoma cell lines. The outcome of this experiment can be seen in the lines from 491 to 527.

3) The authors describe viability analysis of tumorspheroids using Alamar Blue™ cell viability reagent. The more commonly used method is the CellTiter-Glo® 3D Cell Viability Assay, based on quantitation of the ATP present. I strongly suggest repetition of the analyses using the latter to perform a side-by-side comparison and to draw meaningful conclusions on the method of choice. At least, representative images on viablity, such fluorescence microscopy are warranted to evaluate viability.

Thank you for the comments. We compared TMZ-induced cytotoxicity using the Alamar Blue™ cell viability assay and the CellTiter-Glo® 3D Cell Viability Assay and demonstrated that both protocols can be used to evaluate 3D cell cytotoxicity. The outcome of this experiment can be seen in the lines from 491 to 527.

4) Experiments on migration/invasion should be incorporated to judge which method is preffered for invasion analyses.

Thank you for the suggestion. However, we are thinking that it is not relevant to conduct experiments on migration/invasion at this point. Since these article types are not expected to include results but may include pilot data.

5) This lab protocol lacks information on organoid formation. The authors should at least mention this in vitro model in the introduction part

Thank you for the idea. Introduction part updated and 3D cell culture to organoid formation can be seen in lines 122 to 129.

6) The material & methods section is incomplete, there is no information on the statistics and applied tests. By contrast, in the results section, every third sentence includes ANOVA.

An extended version of the methodology is included in the current version of the manuscript. Lines 140 to 205 and 248 to 272 show updated methodology.

Minor:

7) There are several grammatical errors throughout the text. Please check carefully and correct grammar. - Done

8) Make size information uniform, i.e. 150µm vs. 150 µm. - Done

9) Introduce abbreviation at its first mention in the text, e.g. EMT, ANOVA, etc. - Done

10) Generally, revise the text since there are several typing and spelling errors in the current version. - Done

We have edited the document as outlined in points 7-10 above.

**Journal Requirements:**

When submitting your revision, we need you to address these additional requirements.

1. Please ensure that your manuscript meets PLOS ONE's style requirements, including those for file naming. The PLOS ONE style templates can be found at 
https://journals.plos.org/plosone/s/file?id=wjVg/PLOSOne_formatting_sample_main_body.pdf and 
<https://journals.plos.org/plosone/s/file?id=ba62/PLOSOne_formatting_sample_title_authors_affiliations.pdf>.

The manuscript has been updated to meet PLoS One style requirements.

2. Thank you for providing the following Protocols.io DOI in the Methods section of your manuscript 
Low attachment plate method - dx.doi.org/10.17504/protocols.io.bszmnf46
Hanging drop plate method - dx.doi.org/10.17504/protocols.io.btstnnen
Scaffold based method - dx.doi.org/10.17504/protocols.io.bszqnf5w

In keeping with our submission requirements, please add the Protocols.io DOI in the “Protocol DOI” field of the submission form (via “Edit Submission”). For more information, please see our submission guidelines: <https://journals.plos.org/plosone/s/submission-guidelines#loc-guidelines-for-specific-study-types>.

There are three DOI links. One has been added to the protocol doi field of the submission form. We have included DOI links alongside each method in the methods section.

3. Please provide additional details regarding participant consent. In the ethics statement in the Methods and online submission information, please ensure that you have specified (1) whether consent was informed and (2) what type you obtained (for instance, written or verbal, and if verbal, how it was documented and witnessed). If your study included minors, state whether you obtained consent from parents or guardians. If the need for consent was waived by the ethics committee, please include this information.

If you are reporting a retrospective study of medical records or archived samples, please ensure that you have discussed whether all data were fully anonymized before you accessed them and/or whether the IRB or ethics committee waived the requirement for informed consent. If patients provided informed written consent to have data from their medical records used in research, please include this information.

Not relevant.

4. Thank you for stating the following in the Acknowledgments Section of your manuscript: 
[This study was supported by Science Foundation Ireland (SFI) under Grant Number 17/CDA/4653 and funded through Teagasc Walsh Fellowship. The authors also thank TU Dublin, ESHI, and FOCAS Research Institutes for the use of facilities and support of technical staff.]

We note that you have provided funding information that is not currently declared in your Funding Statement. However, funding information should not appear in the Acknowledgments section or other areas of your manuscript. We will only publish funding information present in the Funding Statement section of the online submission form. 
Please remove any funding-related text from the manuscript and let us know how you would like to update your Funding Statement. Currently, your Funding Statement reads as follows: 
 [Science Foundation Ireland (SFI) Grant Number 17/CDA/4653 (BT, PJC, JC)
Teagasc Walsh Fellowship 2017228 (JMW)].
  
Please state what role the funders took in the study. If the funders had no role, please state: "The funders had no role in study design, data collection and analysis, decision to publish, or preparation of the manuscript." 
If this statement is not correct you must amend it as needed.

Please include this amended Role of Funder statement in your cover letter; we will change the online submission form on your behalf.

Funding information has been removed from the Acknowledgments section in the manuscript and included in the funding statement in the online submission form.

5. We note that you have indicated that data from this study are available upon request. PLOS only allows data to be available upon request if there are legal or ethical restrictions on sharing data publicly. For information on unacceptable data access restrictions, please see http://journals.plos.org/plosone/s/data-availability#loc-unacceptable-data-access-restrictions.

In your revised cover letter, please address the following prompts:

a) If there are ethical or legal restrictions on sharing a de-identified data set, please explain them in detail (e.g., data contain potentially identifying or sensitive patient information) and who has imposed them (e.g., an ethics committee). Please also provide contact information for a data access committee, ethics committee, or other institutional body to which data requests may be sent.

b) If there are no restrictions, please upload the minimal anonymized data set necessary to replicate your study findings as either Supporting Information files or to a stable, public repository and provide us with the relevant URLs, DOIs, or accession numbers. Please see http://www.bmj.com/content/340/bmj.c181.long for guidelines on how to de-identify and prepare clinical data for publication. For a list of acceptable repositories, please see http://journals.plos.org/plosone/s/data-availability#loc-recommended-repositories.

We will update your Data Availability statement on your behalf to reflect the information you provide.

There are no ethical or legal restrictions to share data sets. We will upload all the relevant data sets as a Supporting Information files.

6. Please include your full ethics statement in the ‘Methods’ section of your manuscript file. In your statement, please include the full name of the IRB or ethics committee who approved or waived your study, as well as whether or not you obtained informed written or verbal consent. If consent was waived for your study, please include this information in your statement as well.

Not relevant to this study since this research is not using any human subjects or samples. This project is also not involving any primary cell lines. All Research in TU Dublin must be approved by the TU Dublin Research Ethics and Integrity Committee (REIC) and this approval was sought and granted prior to commencing the study.
